# Supplementary material for: The Effects of Complementary Therapies on Patient-Reported Outcomes: An Overview of Recent Systematic Reviews in Oncology
Source: Cancers (Basel). 2023 Sep 11;15(18):4513. doi: 10.3390/cancers15184513 (PMC10526744; doi:10.3390/cancers15184513)
Supplement: Supplementary file 1 [file cancers-15-04513-s001.zip › Material S1 - Search terms final version.pdf]

Supplemental material S1 – search terms

Search term Pubmed

("neoplasms "[MeSH Terms] OR neoplasm*[tiab] OR cancer*[tiab] OR tumor*[tiab] OR tumour*[tiab] OR carcinoma*[tiab]) AND ("integrative medicine"[MeSH Terms] OR integrative medicine[tiab] OR integrative oncol*[tiab] OR "complementary therapies"[MeSH Terms] OR complementary therap*[tiab] OR complementary medicine[tiab] OR complementary modalit*[tiab] OR complementary and alternative medicine[tiab] OR CAM[tiab] OR CIM[tiab] OR Acupuncture[tiab] OR Electroacupuncture[tiab] OR Meridian*[tiab] OR Moxibustion[tiab] OR Anthroposoph*[tiab] OR Auriculotherap*[tiab] OR Cupping Therap*[tiab] OR Diffuse Noxious Inhibitory Control[tiab] OR Dry Needling[tiab] OR Holistic Health[tiab] OR Holistic Therap*[tiab] OR Holistic Medicine[tiab] OR Bioresonance Therap*[tiab] OR Homeopathy[tiab] OR Horticultural Therap*[tiab] OR Gardening Therap*[tiab] OR Traditional Medicine[tiab] OR Home Remed*[tiab] OR Traditional Chinese Medicine[tiab] OR Ayurvedic Medicine[tiab] OR Ayurveda[tiab] OR Oriental Medicine[tiab] OR Mesotherap*[tiab] OR mind body medicine[tiab] OR mind body therap*[tiab] OR Aromatherap*[tiab] OR Biofeedback[tiab] OR Neurofeedback[tiab] OR Breathing Exercise*[tiab] OR Qigong[tiab] OR Hypnosis[tiab] OR Hypnotherap*[tiab] OR Suggestion[tiab] OR Autogenic Training*[tiab] OR Imagery[tiab] OR Guided Imagery[tiab] OR Laughter Therap*[tiab] OR Meditation[tiab] OR Mental Healing*[tiab] OR Mindfulness[tiab] OR Mindfulness based stress reduction[tiab] OR MBSR[tiab] OR Psychodrama[tiab] OR Role Playing[tiab] OR Tai Ji[tiab] OR Tai Chi[tiab] OR Therapeutic Touch[tiab] OR Yoga[tiab] OR Musculoskeletal Manipulation*[tiab] OR Manipulative Therap*[tiab] OR Manipulation Therap*[tiab] OR Manual Therap*[tiab] OR Reflexology[tiab] OR Kinesiology[tiab] OR Chiropractic Manipulation*[tiab] OR Chiropractic Adjustment*[tiab] OR Osteopathic Manipulati*[tiab] OR Acupressure[tiab] OR Shiatsu[tiab] OR Massage*[tiab] OR Massage Therap*[tiab] OR Zone Therap*[tiab] OR Manual Lymphatic Drainage[tiab] OR Manual Lymph Drainage[tiab] OR Myofascial Release Therap*[tiab] OR Naturopath*[tiab] OR Naturopathic Medicine[tiab] OR Phytotherap*[tiab] OR Herb Medicine[tiab] OR Herbal therap*[tiab] OR Herbal Medicine[tiab] OR Aromatherap*[tiab] OR Reflexotherap*[tiab] OR Reflex therap*[tiab] OR Sensory Art Therap*[tiab] OR Acoustic Stimulation[tiab] OR Auditory Stimulation[tiab] OR Art Therap*[tiab] OR Color Therap*[tiab] OR Dance Therap*[tiab] OR Music Therap*[tiab] OR Play Therap*[tiab] OR Spiritual Therap*[tiab] OR Faith Healing[tiab] OR Meditation[tiab] OR Relaxation[tiab] OR Mental Healing[tiab] OR Therapeutic Touch[tiab] OR Energetic Medicine[tiab] OR Touch therap*[tiab] OR Reiki[tiab] OR Yoga[tiab]) AND ((systematic* [ti] AND review [ti]) OR Systematic overview* [ti] OR Cochrane review* [ti] OR systemic review* [ti] OR scoping review [ti] OR scoping literature review [ti] OR mapping review [ti] OR Umbrella review* [ti] OR (review of reviews [ti] OR overview of reviews [ti]) OR meta-review [ti] OR (integrative review [ti] OR integrated review [ti] OR integrative overview [ti] OR meta-synthesis [ti] OR metasynthesis [ti] OR quantitative review [ti] OR quantitative synthesis [ti] OR research synthesis [ti] OR meta-ethnography [ti]) OR Systematic literature search [ti] OR Systematic literature research [ti] OR meta-analyses [ti] OR metaanalyses [ti] OR metaanalysis [ti] OR meta-analysis [ti] OR meta-analytic review [ti] OR meta-analytical review [ti] OR meta-analysis [pt] OR ((search* [tiab] OR medline [tiab] OR pubmed [tiab] OR embase [tiab] OR Cochrane [tiab] OR scopus [tiab] or web of science [tiab] OR sources of information [tiab] OR data sources [tiab] OR following databases [tiab]) AND (study selection [tiab] OR selection criteria [tiab] OR eligibility criteria [tiab] OR inclusion criteria [tiab] OR exclusion criteria [tiab])) OR systematic review [pt]) NOT (letter [pt] OR editorial [pt] OR comment [pt] OR case reports [pt] OR historical article [pt] OR report [ti] OR protocol [ti] OR protocols [ti] OR withdrawn [ti] OR retraction of publication [pt] OR retraction of publication as topic [mesh] OR retracted publication [pt] OR reply [ti] OR published erratum [pt])

AND (english[Language]) AND ("2018/01/01"[Date - Publication] : "2022/04/20 "[Date - Publication])

Search term Embase

(exp neoplasm/ OR neoplasm*.ti,ab,kf. OR cancer*.ti,ab,kf. OR tumor*.ti,ab,kf. OR tumour*.ti,ab,kf. OR carcinoma*.ti,ab,kf.) AND (exp "integrative medicine "/ OR "integrative medicine".ti,ab,kf. OR "integrative oncol*".ti,ab,kf. OR exp "alternative medicine"/ OR "complementary therap*".ti,ab,kf. OR "complementary medicine".ti,ab,kf. OR "complementary modalit*".ti,ab,kf. OR "complementary and alternative medicine".ti,ab,kf. OR CAM.ti,ab,kf. OR CIM.ti,ab,kf. OR Acupuncture.ti,ab,kf. OR Electroacupuncture.ti,ab,kf. OR Meridian*.ti,ab,kf. OR Moxibustion.ti,ab,kf. OR Anthroposoph*.ti,ab,kf. OR Auriculotherap*.ti,ab,kf. OR "Cupping Therap*".ti,ab,kf. OR "Diffuse Noxious Inhibitory Control".ti,ab,kf. OR "Dry Needling".ti,ab,kf. OR "Holistic Health".ti,ab,kf. OR "Holistic Therap*".ti,ab,kf. OR "Holistic Medicine".ti,ab,kf. OR "Bioresonance Therap*".ti,ab,kf. OR Homeopathy.ti,ab,kf. OR "Horticultural Therap*".ti,ab,kf. OR "Gardening Therap*".ti,ab,kf. OR "Traditional Medicine".ti,ab,kf. OR "Home Remed*".ti,ab,kf. OR "Traditional Chinese Medicine".ti,ab,kf. OR "Ayurvedic Medicine".ti,ab,kf. OR Ayurveda.ti,ab,kf. OR "Oriental Medicine".ti,ab,kf. OR Mesotherap*.ti,ab,kf. OR "mind body medicine".ti,ab,kf. OR "mind body therap*".ti,ab,kf. OR Aromatherap*.ti,ab,kf. OR Biofeedback.ti,ab,kf. OR Neurofeedback.ti,ab,kf. OR "Breathing Exercise*".ti,ab,kf. OR Qigong.ti,ab,kf. OR Hypnosis.ti,ab,kf. OR Hypnotherap*.ti,ab,kf. OR Suggestion.ti,ab,kf. OR "Autogenic Training*".ti,ab,kf. OR Imagery.ti,ab,kf. OR "Guided Imagery".ti,ab,kf. OR "Laughter Therap*".ti,ab,kf. OR Meditation.ti,ab,kf. OR "Mental Healing*".ti,ab,kf. OR Mindfulness.ti,ab,kf. OR "Mindfulness based stress reduction".ti,ab,kf. OR MBSR.ti,ab,kf. OR Psychodrama.ti,ab,kf. OR "Role Playing".ti,ab,kf. OR "Tai Ji".ti,ab,kf. OR "Tai Chi".ti,ab,kf. OR "Therapeutic Touch".ti,ab,kf. OR Yoga.ti,ab,kf. OR "Musculoskeletal Manipulation*".ti,ab,kf. OR "Manipulative Therap*".ti,ab,kf. OR "Manipulation Therap*".ti,ab,kf. OR "Manual Therap*".ti,ab,kf. OR Reflexology.ti,ab,kf. OR Kinesiology.ti,ab,kf. OR "Chiropractic Manipulation*".ti,ab,kf. OR "Chiropractic Adjustment*".ti,ab,kf. OR "Osteopathic Manipulati*".ti,ab,kf. OR Acupressure.ti,ab,kf. OR Shiatsu.ti,ab,kf. OR Massage*.ti,ab,kf. OR "Massage Therap*".ti,ab,kf. OR "Zone Therap*".ti,ab,kf. OR "Manual Lymphatic Drainage".ti,ab,kf. OR "Manual Lymph Drainage".ti,ab,kf. OR "Myofascial Release Therap*".ti,ab,kf. OR Naturopath*.ti,ab,kf. OR "Naturopathic Medicine".ti,ab,kf. OR Phytotherap*.ti,ab,kf. OR "Herb Medicine".ti,ab,kf. OR "Herbal therap*".ti,ab,kf. OR "Herbal Medicine".ti,ab,kf. OR Aromatherap*.ti,ab,kf. OR Reflexotherap*.ti,ab,kf. OR "Reflex therap*".ti,ab,kf. OR "Sensory Art Therap*".ti,ab,kf. OR "Acoustic Stimulation".ti,ab,kf. OR "Auditory Stimulation".ti,ab,kf. OR "Art Therap*".ti,ab,kf. OR "Color Therap*".ti,ab,kf. OR "Dance Therap*".ti,ab,kf. OR "Music Therap*".ti,ab,kf. OR "Play Therap*".ti,ab,kf. OR "Spiritual Therap*".ti,ab,kf. OR "Faith Healing".ti,ab,kf. OR Meditation.ti,ab,kf. OR Relaxation.ti,ab,kf. OR "Mental Healing".ti,ab,kf. OR "Therapeutic Touch".ti,ab,kf. OR "Energetic Medicine".ti,ab,kf. OR "Touch therap*".ti,ab,kf. OR Reiki.ti,ab,kf. OR Yoga.ti,ab,kf.) AND ((systematic*.ti. AND review.ti.) OR "Systematic overview*".ti. OR "Cochrane review*".ti. OR "systemic review*".ti. OR "scoping review".ti. OR "scoping literature review".ti. OR "mapping review".ti. OR "Umbrella review*".ti. OR ("review of reviews".ti. OR "overview of reviews".ti.) OR meta-review.ti. OR ("integrative review".ti. OR "integrated review".ti. OR "integrative overview".ti. OR meta-synthesis.ti. OR metasynthesis.ti. OR "quantitative review".ti. OR "quantitative synthesis".ti. OR "research synthesis".ti. OR meta-ethnography.ti.) OR "Systematic literature search".ti. OR "Systematic literature research".ti. OR meta-analyses.ti. OR metaanalyses.ti. OR metaanalysis.ti. OR meta-analysis.ti. OR "meta-analytic review".ti. OR "meta-analytical review".ti. OR meta-analysis.pt. OR ((search*.ti,ab,kf. OR medline.ti,ab,kf. OR pubmed.ti,ab,kf. OR embase.ti,ab,kf . OR Cochrane.ti,ab,kf. OR scopus.ti,ab,kf. OR "web of science".ti,ab,kf. OR "sources

of information".ti,ab,kf. OR "data sources".ti,ab,kf. OR "following databases".ti,ab,kf.) AND ("study selection".ti,ab,kf. OR "selection criteria".ti,ab,kf. OR "eligibility criteria".ti,ab,kf. OR "inclusion criteria".ti,ab,kf. OR "exclusion criteria".ti,ab,kf.)) OR "systematic review"/) NOT (letter.pt. OR editorial.ti. OR "conference abstract".pt. OR "conference paper".pt. OR "conference review".pt. OR comment.ti. OR "case reports".ti. OR "historical article".ti. OR report.ti. OR protocol.ti. OR protocols.ti. OR withdrawn.ti. OR tombstone.pt. OR "retraction of publication".ti. OR exp "retraction of publication as topic" OR "retracted publication".ti. OR reply.ti. OR "erratum".pt.)

Manual filter: language (English) and publication date (from 01 January 2018 till 20 April 2022)

Search term PsycINFO

(exp neoplasms/ OR neoplasm*.ti,ab,id. OR cancer*.ti,ab,id. OR tumor*.ti,ab,id. OR tumour*.ti,ab,id. OR carcinoma*.ti,ab,id.) AND (e xp "alternative medicine"/ OR "integrative medicine".ti,ab,id. OR "integrative oncol*".ti,ab,id. OR "complementary therapies" OR "complementary therap*".ti,ab,id. OR "complementary medicine".ti,ab,id. OR "complementary modalit*".ti,ab,id. OR "complementary and alternative medicine".ti,ab,id. OR CAM.ti,ab,id. OR CIM.ti,ab,id. OR Acupuncture.ti,ab,id. OR Electroacupuncture.ti,ab,id. OR Meridian*.ti,ab,id. OR Moxibustion.ti,ab,id. OR Anthroposoph*.ti,ab,id. OR Auriculotherap*.ti,ab,id. OR "Cupping Therap*".ti,ab,id. OR "Diffuse Noxious Inhibitory Control".ti,ab,id. OR "Dry Needling".ti,ab,id. OR "Holistic Health".ti,ab,id. OR "Holistic Therap*".ti,ab,id. OR "Holistic Medicine".ti,ab,id. OR "Bioresonance Therap*".ti,ab,id. OR Homeopathy.ti,ab,id. OR "Horticultural Therap*".ti,ab,id. OR "Gardening Therap*".ti,ab,id. OR "Traditional Medicine".ti,ab,id. OR "Home Remed*".ti,ab,id. OR "Traditional Chinese Medicine".ti,ab,id. OR "Ayurvedic Medicine".ti,ab,id. OR Ayurveda.ti,ab,id. OR "Oriental Medicine".ti,ab,id. OR Mesotherap*.ti,ab,id. OR "mind body medicine".ti,ab,id. OR "mind body therap*".ti,ab,id. OR Aromatherap*.ti,ab,id. OR Biofeedback.ti,ab,id. OR Neurofeedback.ti,ab,id. OR "Breathing Exercise*".ti,ab,id. OR Qigong.ti,ab,id. OR Hypnosis.ti,ab,id. OR Hypnotherap*.ti,ab,id. OR Suggestion.ti,ab,id. OR "Autogenic Training*".ti,ab,id. OR Imagery.ti,ab,id. OR "Guided Imagery".ti,ab,id. OR "Laughter Therap*".ti,ab,id. OR Meditation.ti,ab,id. OR "Mental Healing*".ti,ab,id. OR Mindfulness.ti,ab,id. OR "Mindfulness based stress reduction".ti,ab,id. OR MBSR.ti,ab,id. OR Psychodrama.ti,ab,id. OR "Role Playing".ti,ab,id. OR "Tai Ji".ti,ab,id. OR "Tai Chi".ti,ab,id. OR "Therapeutic Touch".ti,ab,id. OR Yoga.ti,ab,id. OR "Musculoskeletal Manipulation*".ti,ab,id. OR "Manipulative Therap*".ti,ab,id. OR "Manipulation Therap*".ti,ab,id. OR "Manual Therap*".ti,ab,id. OR Reflexology.ti,ab,id. OR Kinesiology.ti,ab,id. OR "Chiropractic Manipulation*".ti,ab,id. OR "Chiropractic Adjustment*".ti,ab,id. OR "Osteopathic Manipulati*".ti,ab,id. OR Acupressure.ti,ab,id. OR Shiatsu.ti,ab,id. OR Massage*.ti,ab,id. OR "Massage Therap*".ti,ab,id. OR "Zone Therap*".ti,ab,id. OR "Manual Lymphatic Drainage".ti,ab,id. OR "Manual Lymph Drainage".ti,ab,id. OR "Myofascial Release Therap*".ti,ab,id. OR Naturopath*.ti,ab,id. OR "Naturopathic Medicine".ti,ab,id. OR Phytotherap*.ti,ab,id. OR "Herb Medicine".ti,ab,id. OR "Herbal therap*".ti,ab,id. OR "Herbal Medicine".ti,ab,id. OR Aromatherap*.ti,ab,id. OR Reflexotherap*.ti,ab,id. OR "Reflex therap*".ti,ab,id. OR "Sensory Art Therap*".ti,ab,id. OR "Acoustic Stimulation".ti,ab,id. OR "Auditory Stimulation".ti,ab,id. OR "Art Therap*".ti,ab,id. OR "Color Therap*".ti,ab,id. OR "Dance Therap*".ti,ab,id. OR "Music Therap*".ti,ab,id. OR "Play Therap*".ti,ab,id. OR "Spiritual Therap*".ti,ab,id. OR "Faith Healing".ti,ab,id. OR Meditation.ti,ab,id. OR Relaxation.ti,ab,id. OR "Mental Healing".ti,ab,id. OR "Therapeutic Touch".ti,ab,id. OR "Energetic Medicine".ti,ab,id. OR "Touch therap*".ti,ab,id. OR Reiki.ti,ab,id. OR Yoga.ti,ab,id.) AND ((systematic*.ti. AND review.ti.) OR "Systematic overview*".ti. OR "Cochrane review*".ti. OR "systemic review*".ti. OR "scoping review".ti. OR "scoping literature review".ti. OR "mapping review".ti. OR "Umbrella review*".ti. OR ("review of reviews".ti. OR "overview of reviews".ti.) OR meta-review.ti. OR ("integrative review".ti. OR "integrated review".ti. OR "integrative overview".ti. OR meta-synthesis.ti. OR metasynthesis.ti. OR "quantitative review".ti. OR "quantitative synthesis".ti. OR "research synthesis".ti. OR meta-ethnography.ti.) OR "Systematic literature search".ti. OR "Systematic literature research".ti. OR meta-analyses.ti. OR metaanalyses.ti. OR metaanalysis.ti. OR meta-analysis.ti. OR "meta-analytic review".ti. OR "meta-analytical review".ti. OR meta-analysis.pt. OR ((search*.ti,ab,id. OR medline.ti,ab,id. OR pubmed.ti,ab,id. OR embase.ti,ab,id. OR Cochrane.ti,ab,id. OR scopus.ti,ab,id. OR "web of science".ti,ab,id. OR "sources of information".ti,ab,id. OR "data sources".ti,ab,id. OR "following databases".ti,ab,id.) AND ("study

selection".ti,ab,id. OR "selection criteria".ti,ab,id. OR "eligibility criteria".ti,ab,id. OR "inclusion criteria".ti,ab,id. OR "exclusion criteria".ti,ab,id.)) OR "systematic review".pt.) NOT (letter.ti. OR editorial.ti. OR comment.ti. OR "case reports".ti. OR "historical article".ti. OR report.ti. OR protocol.ti. OR protocols.ti. OR withdrawn.ti. OR "retraction of publication".ti. OR "retraction of publication as topic".ti. OR "retracted publication".ti. OR reply.ti. OR "published erratum".ti.)

Manual filter: language (English) and publication date (from 01 January 2018 till 20 April 2022)

Search term CINAHL

((MH neoplasms+) OR (TI neoplasm* OR AB neoplasm*) OR (TI cancer* OR AB cancer*) OR (TI tumor* OR AB tumor*) OR (TI tumour* OR AB tumour*) OR (TI carcinoma* OR AB carcinoma*)) AND ((MH "integrative medicine"+) OR (TI "integrative medicine" OR AB "integrative medicine") OR (TI "integrative oncol*" OR AB "integrative oncol*") OR (MH "alternative therapies"+) OR (TI "complementary therap*" OR AB "complementary therap*") OR (TI "complementary medicine" OR AB "complementary medicine") OR (TI "complementary modalit*" OR AB "complementary modalit*") OR (TI "complementary and alternative medicine" OR AB "complementary and alternative medicine") OR (TI CAM OR AB CAM) OR (TI CIM OR AB CIM) OR (TI Acupuncture OR AB Acupuncture) OR (TI Electroacupuncture OR AB Electroacupuncture) OR (TI Meridian* OR AB Meridian*) OR (TI Moxibustion OR AB Moxibustion) OR (TI Anthroposoph* OR AB Anthroposoph*) OR (TI Auriculotherap* OR AB Auriculotherap*) OR (TI "Cupping Therap*" OR AB "Cupping Therap*") OR (TI "Diffuse Noxious Inhibitory Control" OR AB "Diffuse Noxious Inhibitory Control") OR (TI "Dry Needling" OR AB "Dry Needling") OR (TI "Holistic Health" OR AB "Holistic Health") OR (TI "Holistic Therap*" OR AB "Holistic Therap*") OR (TI "Holistic Medicine" OR AB "Holistic Medicine") OR (TI "Bioresonance Therap*" OR AB "Bioresonance Therap*") OR (TI Homeopathy OR AB Homeopathy) OR (TI "Horticultural Therap*" OR AB "Horticultural Therap*") OR (TI "Gardening Therap*" OR AB "Gardening Therap*") OR (TI "Traditional Medicine" OR AB "Traditional Medicine") OR (TI "Home Remed*" OR AB "Home Remed*") OR (TI "Traditional Chinese Medicine" OR AB "Traditional Chinese Medicine") OR (TI "Ayurvedic Medicine" OR AB "Ayurvedic Medicine") OR (TI Ayurveda OR AB Ayurveda) OR (TI "Oriental Medicine" OR AB "Oriental Medicine") OR (TI Mesotherap* OR AB Mesotherap*) OR (TI "mind body medicine" OR AB "mind body medicine") OR (TI "mind body therap*" OR AB "mind body therap*") OR (TI Aromatherap* OR AB Aromatherap*) OR (TI Biofeedback OR AB Biofeedback) OR (TI Neurofeedback OR AB Neurofeedback) OR (TI "Breathing Exercise*" OR AB "Breathing Exercise*") OR (TI Qigong OR AB Qigong) OR (TI Hypnosis OR AB Hypnosis) OR (TI Hypnotherap* OR AB Hypnotherap*) OR (TI Suggestion OR AB Suggestion) OR (TI "Autogenic Training*" OR AB "Autogenic Training*") OR (TI Imagery OR AB Imagery) OR (TI "Guided Imagery" OR AB "Guided Imagery") OR (TI "Laughter Therap*" OR AB "Laughter Therap*") OR (TI Meditation OR AB Meditation) OR (TI "Mental Healing*" OR AB "Mental Healing*") OR (TI Mindfulness OR AB Mindfulness) OR (TI "Mindfulness based stress reduction" OR AB "Mindfulness based stress reduction") OR (TI MBSR OR AB MBSR) OR (TI Psychodrama OR AB Psychodrama) OR (TI "Role Playing" OR AB "Role Playing") OR (TI "Tai Ji" OR AB "Tai Ji") OR (TI "Tai Chi" OR AB "Tai Chi") OR (TI "Therapeutic Touch" OR AB "Therapeutic Touch") OR (TI Yoga OR AB Yoga) OR (TI "Musculoskeletal Manipulation*" OR AB "Musculoskeletal Manipulation*") OR (TI "Manipulative Therap*" OR AB "Manipulative Therap*") OR (TI "Manipulation Therap*" OR AB "Manipulation Therap*") OR (TI "Manual Therap*" OR AB "Manual Therap*") OR (TI Reflexology OR AB Reflexology) OR (TI Kinesiology OR AB Kinesiology) OR (TI "Chiropractic Manipulation*" OR AB "Chiropractic Manipulation*") OR (TI "Chiropractic Adjustment*" OR AB "Chiropractic Adjustment*") OR (TI "Osteopathic Manipulati*" OR AB "Osteopathic Manipulati*") OR (TI Acupressure OR AB Acupressure) OR (TI Shiatsu OR AB Shiatsu) OR (TI Massage* OR AB Massage*) OR (TI "Massage Therap*" OR AB "Massage Therap*") OR (TI "Zone Therap*" OR AB "Zone Therap*") OR (TI "Manual Lymphatic Drainage" OR AB "Manual Lymphatic Drainage") OR (TI "Manual Lymph Drainage" OR AB "Manual Lymph Drainage") OR (TI "Myofascial Release Therap*" OR AB "Myofascial Release Therap*") OR (TI Naturopath* OR AB Naturopath*) OR (TI "Naturopathic Medicine" OR AB "Naturopathic Medicine") OR (TI Phytotherap* OR AB Phytotherap*) OR (TI "Herb Medicine" OR AB "Herb Medicine") OR (TI "Herbal therap*" OR AB "Herbal therap*") OR (TI "Herbal Medicine" OR AB

"Herbal Medicine") OR (TI Aromatherap* OR AB Aromatherap*) OR (TI Reflexotherap* OR AB Reflexotherap*) OR (TI "Reflex therap*" OR AB "Reflex therap*") OR (TI "Sensory Art Therap*" OR AB "Sensory Art Therap*") OR (TI "Acoustic Stimulation" OR AB "Acoustic Stimulation") OR (TI "Auditory Stimulation" OR AB "Auditory Stimulation") OR (TI "Art Therap*" OR AB "Art Therap*") OR (TI "Color Therap*" OR AB "Color Therap*") OR (TI "Dance Therap*" OR AB "Dance Therap*") OR (TI "Music Therap*" OR AB "Music Therap*") OR (TI "Play Therap*" OR AB "Play Therap*") OR (TI "Spiritual Therap*" OR AB "Spiritual Therap*") OR (TI "Faith Healing" OR AB "Faith Healing") OR (TI Meditation OR AB Meditation) OR (TI Relaxation OR AB Relaxation) OR (TI "Mental Healing" OR AB "Mental Healing") OR (TI "Therapeutic Touch" OR AB "Therapeutic Touch") OR (TI "Energetic Medicine" OR AB "Energetic Medicine") OR (TI "Touch therap*" OR AB "Touch therap*") OR (TI Reiki OR AB Reiki) OR (TI Yoga OR AB Yoga)) AND ((TI systematic* AND TI review) OR TI "Systematic overview*" OR TI "Cochrane review*" OR TI "systemic review*" OR TI "scoping review" OR TI "scoping literature review" OR TI "mapping review" OR TI "Umbrella review*" OR (TI "review of reviews" OR TI "overview of reviews") OR TI meta-review OR (TI "integrative review" OR TI "integrated review" OR TI "integrative overview" OR TI meta-synthesis OR TI metasynthesis OR TI "quantitative review" OR TI "quantitative synthesis" OR TI "research synthesis" OR TI meta-ethnography) OR TI "Systematic literature search" OR TI "Systematic literature research" OR TI meta-analyses OR TI metaanalyses OR TI metaanalysis OR TI meta-analysis OR TI "meta-analytic review" OR TI "meta-analytical review" OR PT meta-analysis OR (((TI search* OR AB search*) OR (TI medline OR AB medline) OR (TI pubmed OR AB pubmed) OR (TI embase OR AB embase) OR (TI Cochrane OR AB Cochrane) OR (TI scopus OR AB scopus) OR (TI "web of science" OR AB "web of science") OR (TI "sources of information" OR AB "sources of information") OR (TI "data sources" OR AB "data sources") OR (TI "following databases" OR AB "following databases")) AND ((TI "study selection" OR AB "study selection") OR (TI "selection criteria" OR AB "selection criteria") OR (TI "eligibility criteria" OR AB "eligibility criteria") OR (TI "inclusion criteria" OR AB "inclusion criteria") OR (TI "exclusion criteria" OR AB "exclusion criteria"))) OR PT "systematic review") NOT (PT letter OR PT editorial OR PT commentary OR PT "case study" OR PT "historical material" OR TI report OR TI protocol OR TI protocols OR TI withdrawn OR TI "retraction of publication" OR TI "retraction of publication as topic" OR TI "published erratum")

Manual filter: language (English) and publication date (from 01 January 2018 till 20 April 2022)

Search term Cochrane

([mh neoplasms] OR neoplasm*:ti,ab OR cancer*:ti,ab OR tumor*:ti,ab OR tumour*:ti,ab OR carcinoma*:ti,ab) AND ([mh "integrative medicine"] OR "integrative medicine":ti,ab OR ("integrative" NEXT oncol*):ti,ab OR [mh "complementary therapies"] OR ("complementary" NEXT therap*):ti,ab OR "complementary medicine":ti,ab OR ("complementary" NEXT modalit*):ti,ab OR "complementary and alternative medicine":ti,ab OR CAM:ti,ab OR CIM:ti,ab OR Acupuncture:ti,ab OR Electroacupuncture:ti,ab OR Meridian*:ti,ab OR Moxibustion:ti,ab OR Anthroposoph*:ti,ab OR Auriculotherap*:ti,ab OR ("Cupping" NEXT Therap*):ti,ab OR "Diffuse Noxious Inhibitory Control":ti,ab OR "Dry Needling":ti,ab OR "Holistic Health":ti,ab OR ("Holistic" NEXT Therap*):ti,ab OR "Holistic Medicine":ti,ab OR ("Bioresonance" NEXT Therap*):ti,ab OR Homeopathy:ti,ab OR ("Horticultural" NEXT Therap*):ti,ab OR ("Gardening" NEXT Therap*):ti,ab OR "Traditional Medicine":ti,ab OR ("Home" NEXT Remed*):ti,ab OR "Traditional Chinese Medicine":ti,ab OR "Ayurvedic Medicine":ti,ab OR Ayurveda:ti,ab OR "Oriental Medicine":ti,ab OR Mesotherap*:ti,ab OR "mind body medicine":ti,ab OR ("mind body" NEXT therap*):ti,ab OR Aromatherap*:ti,ab OR Biofeedback:ti,ab OR Neurofeedback:ti,ab OR ("Breathing" NEXT Exercise*):ti,ab OR Qigong:ti,ab OR Hypnosis:ti,ab OR Hypnotherap*:ti,ab OR Suggestion:ti,ab OR ("Autogenic" NEXT Training*):ti,ab OR Imagery:ti,ab OR "Guided Imagery":ti,ab OR ("Laughter" NEXT Therap*):ti,ab OR Meditation:ti,ab OR ("Mental" NEXT Healing*):ti,ab OR Mindfulness:ti,ab OR "Mindfulness based stress reduction":ti,ab OR MBSR:ti,ab OR Psychodrama:ti,ab OR "Role Playing":ti,ab OR "Tai Ji":ti,ab OR "Tai Chi":ti,ab OR "Therapeutic Touch":ti,ab OR Yoga:ti,ab OR ("Musculoskeletal" NEXT Manipulation*):ti,ab OR ("Manipulative" NEXT Therap*):ti,ab OR ("Manipulation" NEXT Therap*):ti,ab OR ("Manual" NEXT Therap*):ti,ab OR Reflexology:ti,ab OR Kinesiology:ti,ab OR ("Chiropractic" NEXT Manipulation*):ti,ab OR ("Chiropractic" NEXT Adjustment*):ti,ab OR ("Osteopathic" NEXT Manipulati*):ti,ab OR Acupressure:ti,ab OR Shiatsu:ti,ab OR Massage*:ti,ab OR ("Massage" NEXT Therap*):ti,ab OR ("Zone" NEXT Therap*):ti,ab OR "Manual Lymphatic Drainage":ti,ab OR "Manual Lymph Drainage":ti,ab OR ("Myofascial Release" NEXT Therap*):ti,ab OR Naturopath*:ti,ab OR "Naturopathic Medicine":ti,ab OR Phytotherap*:ti,ab OR "Herb Medicine":ti,ab OR ("Herbal" NEXT therap*):ti,ab OR "Herbal Medicine":ti,ab OR Aromatherap*:ti,ab OR Reflexotherap*:ti,ab OR ("Reflex" NEXT therap*):ti,ab OR ("Sensory Art" NEXT Therap*):ti,ab OR "Acoustic Stimulation":ti,ab OR "Auditory Stimulation":ti,ab OR ("Art" NEXT Therap*):ti,ab OR ("Color" NEXT Therap*):ti,ab OR ("Dance" NEXT Therap*):ti,ab OR ("Music" NEXT Therap*):ti,ab OR ("Play" NEXT Therap*):ti,ab OR ("Spiritual" NEXT Therap*):ti,ab OR "Faith Healing":ti,ab OR Meditation:ti,ab OR Relaxation:ti,ab OR "Mental Healing":ti,ab OR "Therapeutic Touch":ti,ab OR "Energetic Medicine":ti,ab OR ("Touch" NEXT therap*):ti,ab OR Reiki:ti,ab OR Yoga:ti,ab)

Manual filter: publication date (from 01 January 2018 till 20 April 2022) and Cochrane reviews
