# Supplementary material for: The Effects of Complementary Therapies on Patient-Reported Outcomes: An Overview of Recent Systematic Reviews in Oncology
Source: Cancers (Basel). 2023 Sep 11;15(18):4513. doi: 10.3390/cancers15184513 (PMC10526744; doi:10.3390/cancers15184513)
Supplement: Supplementary file 1 [file cancers-15-04513-s001.zip › Table S1 - Data extraction table.pdf]

Supplemental material S2 – Data extraction table

Table S1. Data extraction from the included systematic reviews.

**Refer nce**	**Author (year)**	**Aim**	**Participants**	**Intervention**	**Comparat or**	**Outcomes**	**Outcome measures**	**Nr of RCTs for outco me**	**Total nr of participants (intervention/c ontrol)**	**Effect size (95% CI), heterogenit y**	**Signific ancy (P-value)**	**Overall conclusion**	***Comment (by Marit Mentink)***
[1]	Armer et al (2022)	To examine effects of yoga on pre- to postintervent ion improvement s in fatigue among cancer patients	Cancer survivors	Yoga	Waitlist, standard care, and active compariso n	Fatigue	Various (FACT, BFI, FSI)	29	1828	Hedges g=0.45, 95%CI: [0.09, 0.82], I^2=90.87%	+ P= 0.013	Yoga may be beneficial as a component of treatment for both fatigue and depression in cancer survivors	*Yoga interventions not significant when compared with active control. Yoga type significant moderator*
						Depression	Various (FACT, EORTC)	12	N/R	Hedges g=0.72, 95%CI: [0.20, 1.24], I^2=89.82%	+ P= 0.007		*Session length significant moderator*
						Quality of life	Various (BDI, HADS CES)	17	N/R	Hedges g=1.08, 95%CI: [-1.92, 4.07], I^2=99.75%	- P= 0.48		
[2]	Bai et al (2022)	To summarize the quality of life of breast cancer patients treated with integrated treatment method vs. conventional Western medicine (CWM)	Postsurgery breast cancer female patients	Traditional Chinese medicine + CWM	CWM (chemothe rapy, radiothera py, endocrine therapy)	Quality of life	FACT-B, QLQ-BR53, KPS, QLQ-C30	12	N/R	SMD=1.26, 95%CI:[1.12 , 1.40], I^2=59.1%	+ P=0.01	Traditional Chinese medicine, when used in conjunction with the conventiona l Western medicine, could be an effective way in improving the quality of life and	*Chemotherapy with epirubicin exhibited higher quality of life than the chemotherapy without epirubicin among breast cancer patients*

Reference	Author (year)	Aim	Participants	Intervention	Comparator	Outcomes	Outcome measures	Nr of RCTs for outcome	Total nr of participants (intervention/control)	Effect size (95% CI), heterogenity	Significancy (P-value)	Overall conclusion	*Comment (by Marit Mentink)*
												alleviating incidence of associated adverse symptoms	
						Incidence of gastrointestinal adverse reaction		5	N/R	RR=1.33, 95%CI:[1.20, 1.48], I^2=19.5%	+ No p-value reported		
[3]	Bro et al (2018)	To identify the psychological and physical effects of music interventions in cancer treatment	Adults in active cancer treatment	Music interventions	Standard care	Anxiety	Various (STAI, VAS)	9	322/316	SMD=−0.65, 95% CI: [−1.20, −0.11], I^2=90%	+ P=0.02	Music may be a tool in reducing anxiety, pain, and improving mood among patients with cancer in active treatment	*The most effective mode of music intervention appeared to be passive listening to self-selected, recorded music in a single session design*
						Pain	Various (MPQ, VAS)	7	254/254	SMD=−0.88, 95%CI: [−1.45,−0.32], I^2=88%	+ P=0.002		
						Depression	Various (HADS, ZSDS)	2	95/88	SMD=−0.89 ,95% CI: [−2.92 to 1.14], I^2=97%	- P=0.39		
						Distress	Various (NRS, HADS)	2	55/50	SMD=−0.25, 95% CI: [−1.03 to 0.52], I^2=74%	- P=0.05		
						Quality of life	Various (FACT-G, EORTC-QLQ-30)	2	86/55	SMD=−0.21, 95% CI: [−0.55 to 0.14], I^2=0%	- P=0.25		

Reference	Author (year)	Aim	Participants	Intervention	Comparator	Outcomes	Outcome measures	Nr of RCTs for outcome	Total nr of participants (intervention/control)	Effect size (95% CI), heterogenity	Significancy (P-value)	Overall conclusion	*Comment (by Marit Mentink)*
						Mood	Various (POMS, QMS)	4	116/96	SMD=−0.55 , 95% CI: [−0.98 to −0.13], I^2=97%	+ P=0.01		
						Fatigue	Various (VAS, FACIT-F)	3	68/63	SMD=−0.22 95% CI: [−1.08 to 0.63], I^2=80%	- P=0.61		
[4]	Cai et al (2022)	To evaluate the effects of Tai Chi on anxiety and depression symptoms in four chronic conditions: cancer, stroke, heart failure (HF), and chronic obstructive pulmonary disease (COPD)	Patients with cancer	Tai Chi	Regular exercise or usual care	Depressive symptoms	Various (BDI, CES-D, IDS-C, HAMD, SDS)	4	178/157	SMD − 0.63, 95%CI: [−0.42, 0.16], I^2 = 92%	- P=0.12	Tai Chi has a positive effect on anxiety and depression, especially for patients with cancer, stroke, and HF.	*Prescriptions for Tai Chi were inconsistent across the studies. Most studies chose Yang-style or Sun-style Tai Chi.*
						Anxiety	Various (SAS, HAMA)	2	120/115	SMD=−0.69 , 95%CI: [−1.22, −0.17]; I^2=72%	+ P<0.01		
[5]	Chan et al (2021)	To investigate the potential benefits and safety of acupuncture on managing side effects induced by drug therapies in	Participants with breast cancer	Acupuncture (penetrating needles on acupoints)	Sham acupuncture, no treatment, or waitlist control	Gastrointestinal disorders	Nausea level, vomiting level, appetite level, constipation score, diarrhoea score, emesis episodes	6	353/346	SMD=−1.15 ; 95%CI: [−1.65, −0.64], I^2=89%	+ P<0.00001	Beneficial effects of acupuncture in the management of side effects from drug therapies on patients	

Refer nce	Author (year)	Aim	Participants	Intervention	Comparat or	Outcomes	Outcome measures	Nr of RCTs for outco me	Total nr of participants (intervention/c ontrol)	Effect size (95% CI), heterogenit y	Signific ancy (P-value)	Overall conclusion	*Comment (by Marit Mentink)*
		patients with breast cancer										with breast cancer	
						Chemothera py-induced peripheral neuropathy (CIPN)	BPI-SF, FACT-NTX, NPS, PNQ	2	165/164	SMD=-0.56; 95% CI: [−0.80,−0.3 2], I^2=10%	+ P<0.000 01		
						Aromatase Inhibitor-Associated Arthralgia (AIAA)	HAQ-DI, VAS, BPI, WOMAC, M-SACRAH	4	571/373	SMD=−0.39 , 95% CI: [−0.73, −0.05], I^2=82%	+ P=0.02		
						Aromatase Inhibitor-Associated Joint Symptoms	HAQ-DI, WOMAC, M-SACRAH, BPI	3	407/257	SMD=−0.44 ; 95% CI: [−0.79, −0.09], I^2=76%	+ P=0.01		
						Quality of life	FACT, HADS, PROMIS, SAS, SDS, KPS, EORTC-QLQ-C30	9	1029/901	SMD=−0.56 ; 95% CI: [−0.84, −0.27]; I^2=89%	+ P=0.000 1		
[6]	Chang et al (2021)	To evaluate the clinical efficacy of MBSR interventions in the short term	Female breast cancer survivors	Mindfulness based stress reduction (MBSR)		Depression	CES-D, HADS, BDI, ESDS	6	529/495	SMD=−1.32 , 95% CI: [−2.18 to −0.46], I^2=97%	+ P= 0.003	Mindfulness -based stress reduction intervention s are highly beneficial for reducing depression, fatigue, and stress in the short term	*Outcomes measured at end-of-intervention. After 3 months, depression and fatigue were not significantly reduced anymore, but stress level was.*
						Anxiety		3	372/348	SMD=−0.09 , 95% CI: [−0.02 to 0.09]; I^2=0%	- P= 0.47		
						Stress level		3	261/248	SMD=−2.34 , 95% CI;	- P= 0.20		

Refer nce	Author (year)	Aim	Participants	Intervention	Comparat or	Outcomes	Outcome measures	Nr of RCTs for outco me	Total nr of participants (intervention/c ontrol)	Effect size (95% CI), heterogenit y	Signific ancy (P-value)	Overall conclusion	*Comment (by Marit Mentink)*
										[−5.96 to 1.27], I^2=97%			
						Fatigue		4	374/360	SMD=−0.47 ; 95% CI: [−0.59 to −0.34]; I^2=0%	+ P< 0.001		
						Pain		5	423/404	SMD=−0.39 , 95% CI: [−0.81 to 0.03]; I^2=85%	- P= 0.07		
						Sleep quality		5	299/295	SMD=−0.67 , 95% CI: [−1.43 to 0.08]; I^2=94%	- P= 0.06		
						Quality of life		4	371/356	SMD=−0.03 , 95% CI: [−0.37 to 0.31]; I^2=75%	- P= 0.86		
[7]	Chen et al (2018)	To evaluate the available evidence from randomized controlled trials (RCTs) of auricular acupressure (AA) therapy for preventing constipation in leukemia patients undergoing	Patients with leukemia and more than 18 years of age undergoing chemother apy	Auricular acupressure + routine care	Routine care	Constipatio n	Constipation Assessment Scale (CAS)	3	232	MD=−1.51, 95% CI: [−1.89 to −1.14], I^2=0%	+ P<0.01	AA may be recommend ed in addition to routine care including use of laxatives to prevent constipation in leukemia patients undergoing chemothera py	

Reference	Author (year)	Aim	Participants	Intervention	Comparator	Outcomes	Outcome measures	Nr of RCTs for outcome	Total nr of participants (intervention/control)	Effect size (95% CI), heterogenity	Significancy (P-value)	Overall conclusion	*Comment (by Marit Mentink)*
		chemotherapy											
						Health-related quality of life	Patient Assessment of Constipation-Quality of Life (PACQOL)	2	172	MD=–1.28, 95% CI: [–1.44 to –1.13], I^2=0%	+ P<0.01		
[8]	Chen et al (2020)	To evaluate the efficacy and safety of Traditional Chinese Medicine (TCM) combined with chemotherapy for patients with small cell lung cancer	Adult patients diagnosed with small cell lung cancer	Traditional Chinese Medicine (herbs and formulas) + conventional chemotherapy	Conventional chemotherapy	Quality of life	KPS	9	N/R	RR=1.31, 95% CI: [1.21–1.42], I^2=35.5%	+ P<0.001	TCM combined with chemotherapy may improve quality of life	*KPS scores were enhanced in Chinese paten drug subgroup and injection subgroup*
						Gastrointestinal reaction		14	N/R	RR=0.79, 95% CI: [0.71–0.8], I^2=0%	+ P<0.001		*The incidence of gastrointestinal reactions was decreased in Formula subgroup and Chinese paten drug subgroup*
[9]	Chen et al (2021)	To systematically evaluate the clinical effect of auricular acupressure (AA) in prevention and treatment of chemotherapy induced	Patients receiving cancer chemotherapy	Auricular acupressure + antiemetics	Antiemetics	Overall chemotherapy induced nausea and vomiting relief efficiency	N/R	10	442/397	RR=1.31, 95% CI: [1.22 to 1.41], I^2=24%	+ P<0.001	Auricular acupressure supplementation benefited delayed chemotherapy-induced nausea and vomiting as well as constipation, diarrhea,	

Refer nce	Author (year)	Aim	Participants	Intervention	Comparat or	Outcomes	Outcome measures	Nr of RCTs for outco me	Total nr of participants (intervention/c ontrol)	Effect size (95% CI), heterogenit y	Signific ancy (P-value)	Overall conclusion	***Comment (by Marit Mentink)***
		nausea and vomiting										and tiredness. AA alone or AA supplement ation has little effect on acute nausea and acute vomiting.	
						Constipatio n incidence	N/R	6	49/61	RR=0.61, 95% CI: [0.45 to 0.84], I^2=0%	+ P=0.002		
						Diarrhea	N/R	2	33/32	RR=0.66, 95% CI: [0.45 to 0.98], I^2=0%	+ P=0.04		
						Abdominal distension	N/R	2	55/30	RR=0.93, 95% CI: [0.71 to 1.24], I^2=12%	- P=0.63		
						Headache	N/R	2	69/66	RR=0.43, 95% CI: [0.10 to 1.82], I^2=0%	- P=0.25		
						Tiredness	N/R	2	103/102	RR=0.39, 95% CI: [0.26 to 0.58], I^2=0%	+ P<0.000 01		
[10]	Chen et al (2021)	To evaluate the efficacy of traditional herbal medicines (HMs) in alleviating symptoms of	Patients with colorectal cancer	Herbal medicine + chemotherap y	Chemothe rapy	Overall CIGI toxicity	N/R	20	2279/2321	RR=0.78, 95% CI: [0.72 to 0.84], I^2=44%	+ P<0.001	A statistically significant effect of HMs combined with	

Reference	Author (year)	Aim	Participants	Intervention	Comparator	Outcomes	Outcome measures	Nr of RCTs for outcome	Total nr of participants (intervention/control)	Effect size (95% CI), heterogenity	Significancy (P-value)	Overall conclusion	*Comment (by Marit Mentink)*
		Chemotherapy-Induced Gastrointestinal (CIGI) toxicity										chemotherapy on alleviating the overall CIGI toxicity, nausea and vomiting, diarrhea, oral mucositis, or abdominal distension is only shown in studies without a double-blind design	
						Nausea and vomiting	N/R	17	909/925	RR=0.74, 95% CI: [0.66 to 0.82], I^2=35%	+ P<0.001		
						Diarrhea	N/R	13	560/571	RR=0.64, 95% CI: [0.44 to 0.93], I^2=50%	+ P=0.02		
						Oral mucositis	N/R	5	231/235	RR=0.65, 95% CI: [0.48 to 0.88], I2=24%	+ P=0.005		
						Abdominal distension	N/R	5	167/171	RR=0.36, 95% CI: [0.18–0.73], I^2=0%	+ P = 0.004		
						Anorexia	N/R	7	275/281	RR=0.75, 95% CI: [0.50–1.11], I^2=67%	- P=0.15		

Reference	Author (year)	Aim	Participants	Intervention	Comparator	Outcomes	Outcome measures	Nr of RCTs for outcome	Total nr of participants (intervention/control)	Effect size (95% CI), heterogenity	Significancy (P-value)	Overall conclusion	***Comment (by Marit Mentink)***
						Constipation	N/R	3	137/142	RR=0.76, 95% CI: [0.45–1.28], I2=0%	- P=0.30		
						Abdominal pain	N/R	3	101/105	RR=0.43, 95% CI: [0.09–1.99], I^2 = 5%	- P=0.35		
[11]	Chen et al (2019)	To systematically review and evaluate the effectiveness of Chinese herbal medicine (CHM) therapy for epidermal growth factor receptor inhibitor (EGFRI)-induced skin rash in patients with malignancy	The patients were diagnosed as malignant tumor, received EGFRIs, and experienced skin rash	Third rule of TCM	Western medicine	Improving skin rash	CTCAE, MASCC	9	333/284	RR=1.70, 95%CI: [1.31 to 2.19], I^2=83%	+ P<0.0001	The limited evidence suggests that CHM exhibits clinical effectiveness and good safety on the treatment of EGFRI-induced skin rash	*Curative efficiency on skin rash in the third rule of TCM is also higher than that of the western medicine group and blank control group*
					Blank control			4	164/119	RR=2.37, 95% CI: [1.21 to 4.63], I^2=89%	+ P<0.00001		
[12]	Chien et al (2019)	To assess the efficacy of acupuncture in treating chemotherapy-induced peripheral neuropathy (CIPN)	Patients afflicted with chemotherapy-induced peripheral neuropathy (CIPN)	True needle or electroacupuncture	Blank control, sham acupuncture, usual care, vitamin B or methyl cobalamin	Pain	Brief Pain Inventory–Short Form worst pain score (BPI-SF)	3	117/112	MD=−1.21, 95% CI: [−1.61 to −0.82], I^2=89%	+ P<0.00001	Acupuncture can effectively relieve CIPN pain and functional limitation	

Refer nce	Author (year)	Aim	Participants	Intervention	Comparat or	Outcomes	Outcome measures	Nr of RCTs for outco me	Total nr of participants (intervention/c ontrol)	Effect size (95% CI), heterogenit y	Significancy (P-value)	Overall conclusion	*Comment (by Marit Mentink)*
						Nervous system symptoms	Functional Assessment of Cancer Therapy/Neuro toxicity (FACT-NXT)	4	132/129	MD=−2.02, 95% CI: [−2.21 to −1.84], I^2=36%	+ P<0.00001		
[13]	Chien et al (2019)	To evaluate how long the effect of acupuncture on breast cancer-related hot flushes and menopause symptoms lasts	Women with breast cancer	Real or electro-acupuncture	Sham acupuncture, self-care hormone therapy, applied relaxation	Frequency of hot flushes (3m after intervention)	N/A	4	118/96	MD=-1.47, 95% CI: [-4.18 to 1.24], I^2=74%	- P=0.29	Acupuncture significantly alleviated menopause symptoms for at least 3 months, but not hot flushes	
						Severity of hot flushes (3m after intervention)	N/A	2	102/119	MD=-6.08, 95% CI: [-18.62 to 6.47], I^2=94%	- P=0.34		
						Menopausal symptoms (3m after intervention)	Kupperman index	5	184/194	MD=-3.47, 95% CI: [-6.11 to -0.84], I^2=75%	+ P=0.01		
[14]	Coutino et al (2019)	To evaluate if non-pharmacological therapy (NPT) groups are related to a reduction of depressive symptoms among BC patients	Non-metastatic breast cancer	Yoga	No intervention	Depressive symptoms	Various (BDI CES, HADS POMS)	5	168/153	SMD=-0.305, 95% CI: [-0.602 to 0.007]; I^2=41.0%	+ P=0.045	A significant difference emerged for yoga when heterogeneity was reduced	
				Art therapy	No intervention	Depressive symptoms	Various (HADS BDI)	2	280/280	SMD=-0.091; 95% CI: [-0.257	+ P=0.279		

Reference	Author (year)	Aim	Participants	Intervention	Comparator	Outcomes	Outcome measures	Nr of RCTs for outcome	Total nr of participants (intervention/control)	Effect size (95% CI), heterogenity	Significancy (P-value)	Overall conclusion	*Comment (by Marit Mentink)*
										to 0.074]; I^2=0.0%			
[15]	Dai et al (2021)	To comprehensively evaluate the effectiveness of acupuncture and derived therapies for analgesia in palliative cancer care	Adults with cancer	Manual acupuncture	Conventional analgesics	Change in pain intensity	NRS	7	438	WMD: 0.90, 95% CI: [0.64 to 1.15]	+ P<0.001	This systematic review supported the application of acupuncture and derived therapies for managing pain during palliative cancer care	*LI4 (Hegu) was the most frequently used acupoint. Asian patients may respond better than Western patients to acupuncture therapy.*
				Electronic acupuncture		Change in pain intensity	NRS	7	454	WMD:1.18, 95% CI: [0.63 to 1.72]	+ P<0.001		
[16]	Danon et al (2021)	To assess whether mind–body therapies are effective for relieving cancer-related pain in adults	Adults with cancer-related pain	Yoga		Pain		7	250/222	SMD:-0.30, 95% CI: [-0.73 to 0.13], I^2=80.0%	- P-value not reported	No specific conclusion for yoga	
[17]	Deng et al (2018)	To evaluate the efficacy of herbal medicine for the prevention and management of hand–foot syndrome	Adult patients clearly diagnosed malignant with a treatment of fluoropyrimidine-based	Herbal medicine interventions (single herb, a compound of several herbs and extracts)	Placebo/blank control, conventional management such as VB6	Quality of life	KPS	8	732	RR=1.58, 95% CI: [1.34, 1.87], I^2=0%	+ P<0.01	The addition of herbal medicine significantly reduced the incidences of all-grade and high-grade HFS and	

Reference	Author (year)	Aim	Participants	Intervention	Comparator	Outcomes	Outcome measures	Nr of RCTs for outcome	Total nr of participants (intervention/control)	Effect size (95% CI), heterogenity	Significancy (P-value)	Overall conclusion	*Comment (by Marit Mentink)*
		(HFS) induced by fluoropyrimidines	chemotherapy									improved patients' QOL	
							QLQ-C30	4	326	MD=9.88, 95% CI: [5.03, 14.73], I^2=87%	+ P<0.01		
						Hand foot syndrome (all grade)		21	889/846	RR=0.64, 95% CI: [0.54-0.76], I^2=44%	+ P<0.00001		
[18]	Dong et al (2019)	To critically evaluate the effect of yoga on cancer-related fatigue in patients with breast cancer	Adult patients diagnosed with breast cancer	Yoga	Any type (standard care, non-intervention, exercise)	Fatigue	Various (FACIT, EORTC-QOL-C30, FSI, VAS)	18	1112/1071	SMD=−0.31, 95% CI: [−0.52, −0.10], I^2=81%	+ P=0.003	Yoga can be considered as an alternative therapy for relieving fatigue in breast cancer patients	*Supervised yoga-class demonstrated a large beneficial effect, while supervised yoga + home based yoga had no effect*
[19]	Dong et al (2021)	To critically assess the effect of wrist-ankle acupuncture (WAA) on cancer pain	Adult patients who were diagnosed with cancer	Wrist-ankle acupuncture with our without analgesics	Analgesics	Pain relief rate	N/A	12	694/641	RR=1.31, 95%CI: [1.15–1.49], I^2=57%	+ P<0.01	WAA has certain effect on cancer pain, and the effect of WAA combined with pharmacological intervention is better than that of drug therapy alone	*The pain relief rate of WAA plus drug therapy group was significantly higher than that of control group*

Refer nce	Author (year)	Aim	Participants	Intervention	Comparat or	Outcomes	Outcome measures	Nr of RCTs for outco me	Total nr of participants (intervention/c ontrol)	Effect size (95% CI), heterogenit y	Signific ancy (P-value)	Overall conclusion	*Comment (by Marit Mentink)*
						Pain score	VAS, NRS	5	230/236	SMD=−0.91 , 95%CI: [−1.70 to −0.13], I^2=93%	+ P = 0.02		
[20]	El-Hashi mi & Gorey (2019)	To explore the hypothesis that yoga is more effective in enhancing the quality of life of women with breast cancer than are other types of exercise	Nonmetast asized breast cancer (including survivors)	Yoga	Any exercise interventio n	Quality of life (postinterve ntion)	Various (BFI, CES-D, SF-36, FACT-B, CFS-D)	8	272/273	Sample weighted d-index=0.14, 90%CI:[0.00 to 0.28]	- P=0.10	Yoga is probably as effective as other exercise modalities in improving the QoL of women with breast cancer.	
						Quality of life (follow up)	Various (BFI, CES-D, SF-36, FACT-B, CFS-D)	8	221/219	Sample weighted d-index=-0.05, 90%CI:[-0.21 to 0.11]	- P-value not reporte d		
[21]	Gao et al (2021)	To evaluate the effects of acupuncture and moxibustion (AM) in women with breast cancer-related lymphedema (BCRL)	Women with lymphedem a, which was caused by BC and its treatment procedures	Acupuncture + functional exercises	Functional exercises	Pain	VAS	2	60/60	MD=−1.33, 95% CI: [−1.52 to −1.15], I^2=0%	+ P < 0.00001	AM is effective in the treatment of BCRL	
				Moxibustion	Pneumatic circulation	Swelling	VAS	3	58/56	MD=−0.51, 95%	+ P=0.003		

Reference	Author (year)	Aim	Participants	Intervention	Comparator	Outcomes	Outcome measures	Nr of RCTs for outcome	Total nr of participants (intervention/control)	Effect size (95% CI), heterogenity	Significancy (P-value)	Overall conclusion	*Comment (by Marit Mentink)*
										CI: [−0.85 to −0.17], I^2=0%			
[22]	Gonzalez et al (2021)	To examine the effects of asana-based yoga interventions on self-reported depression and anxiety symptoms, across a number of cancers	Adult with diagnosis of any cancer (active treatment or post-treatment)	Asana-based yoga interventions	Active control, wait list, usual care	Depressive symptoms	Various (BDI, HADS, POMS)	25	Total = 1486	g=−0.419, 95% CI: [−0.558 to −0.281], I^2=36.91%	+ P<0.001	In people with cancer, yoga-based interventions are associated with amelioration of depression and anxiety symptoms and therefore a promising therapeutic modality for their management	
						Anxiety	Various (HADS, BAI, STAI)	15	Total = 977	g=−0.347, 95% CI: [−0.473 to −0.221], I^2=0%	+ P<0.001		*Subgroup analysis revealed no significant effect in participants who had completed cancer treatment*
[23]	Hausmann et al (2022)	To analyze the general efficacy of yoga, psychosocial, and mindfulness-based interventions for CRF	Adult cancer patients with current or previous curative systemic or radiation therapy	Yoga interventions	Any kind	Cancer-related fatigue (post intervention)	Various (e.g. FACT-B, BFI, FACIT-F, EORTC-QLQ-C30)	24	890/836	SMD=−0.35, 95% CI :[−0.52, −0.19], I^2=61%	P<0.001	Yoga is an effective therapeutic measure to reduce CRF	*No specific intervention characteristic emerged as being more essential or advantageous than others*

Reference	Author (year)	Aim	Participants	Intervention	Comparator	Outcomes	Outcome measures	Nr of RCTs for outcome	Total nr of participants (intervention/control)	Effect size (95% CI), heterogenity	Significancy (P-value)	Overall conclusion	*Comment (by Marit Mentink)*
[24]	Jin et al (2021)	To explore the survival benefits of Traditional Herbal Medicine (THM) treatment alongside first-line platinum-based chemotherapy in patients with non-small-cell lung cancer (NSCLC)	Patients diagnosed with advanced NSCLC	Traditional herbal medicine + platinum-based chemotherapy treatment (PBCT)	PBCT	Nausea and vomiting (grades 3 to 4) after treatment	N/R	5	203/201	RR=0.76, 95% CI [0.66, 0.86], I^2=90%	+ P<0.001	THM was effective in reducing chemotherapy-related adverse effects, including nausea/vomiting.	
[25]	He et al (2020)	To systematically evaluate the evidence for mindfulness-based stress reduction (MBSR) in cancer related fatigue (CRF).	Patients which have been diagnosed as cancer meeting the diagnostic criteria for cancer-induced fatigue.	MBSR	Routine treatment	Cancer related fatigue	Various (CFS, PFS, FSI)	5	356/344	SMD=-0.51, 95%CI: [-0.81 to -0.20], I^2=69%	+ P=0.001	Mindfulness decompression therapy can alleviate cancer-related fatigue to a certain extent	
[26]	He et al (2020)	To evaluate the existing randomized clinical trials (RCTs) for evidence of the association of acupuncture and acupressure with	Patients with cancer	Acupuncture (manual, electro and auricular)	Sham	Pain intensity	BPI, NRS, VAS, VRS	7	226/172	MD=−1.38; 95% CI: [−2.13 to −0.64]; I^2=81%	+ P-value not reported	Acupuncture and/or acupressure was significantly associated with reduced cancer pain, although the evidence	

Reference	Author (year)	Aim	Participants	Intervention	Comparator	Outcomes	Outcome measures	Nr of RCTs for outcome	Total nr of participants (intervention/control)	Effect size (95% CI), heterogenity	Significancy (P-value)	Overall conclusion	*Comment (by Marit Mentink)*
		reduction in cancer pain										level was moderate	
				Acupuncture (manual, electro and auricular)	Wait list	Pain intensity		3		MD=−1.63; 95% CI: [−2.14 to −1.13]; I^2=0%	+ P-value not reported		
[27]	Hou & Chen (2020)	To evaluate the efficacy of acupuncture combined with opiates in the treatment of cancer pain	Patients had to meet the diagnostic criteria on cancer pain	Acupuncture (electro, ear, wrist and ankle, floating needle) + opiates	Opiates	Total effect on cancer pain	NRS, bursts of pain	18	N/R	OR=2.98, 95% CI: [2.38 to 3.73], I^2=0%	+ P<0.05	Acupuncture combined with opioids for cancer pain is superior to opioids alone with a lower incidence of adverse reactions	
						Functional status	KPS	4	96/97	MD=6.12, 95%CI: [1.28-10.97] I^2=68%	+ P=0.01		
[28]	Hsieh et al (2021)	To examine the effect of acupressure on fatigue in cancer survivors	Adults with cancer	Acupressure (manual and auricular)	Sham acupuncture, usual care, active control (acupuncture)	Fatigue	Various (e.g. VAS, BFI, CFS, FACIT-F)	14	Total=776	Hedges g=−0.87. 95% CI: [−1.19 to −0.55], I^2=74.99%	+ P<0.001	Acupressure is effective at alleviating cancer-related fatigue during and after chemotherapy	
[29]	Hsueh et al (2021)	To evaluate the effectiveness of yoga in enhancing the quality of	Patients who had completed breast cancer treatment	Yoga	Usual care, active control (exercise, health education)	Physical well-being	FACT-G, FACT-B	5	114/95	WMD=1.04, 95% CI [-0.03-2.11], I^2=29%	- P=0.06	Yoga may enhance QoL in patients with breast cancer experiencing	*Measurement of global health, functional scales and symptom scales with EORTC QLQ-C30 were not significant*

Refer nce	Author (year)	Aim	Participants	Intervention	Comparat or	Outcomes	Outcome measures	Nr of RCTs for outco me	Total nr of participants (intervention/c ontrol)	Effect size (95% CI), heterogenit y	Significancy (P-value)	Overall conclusion	*Comment (by Marit Mentink)*
		life (QoL) of patients with breast cancer										post-treatment complications.	
						Social well-being		5	114/95	WMD=1.36, 95% CI 0.12–2.61), I^2=38%	+ P=0.03		
						Emotional well-being	FACT-G, FACT-B	5	114/95	WMD=1.46, 95% CI 0.26–2.66], I^2=62%	+ P=0.02		
						Functional well-being	FACT-G, FACT-B	5	114/95	WMD=2.04, 95% CI 0.21–3.87), I^2=70%	+ P=0.03		
						Reduction of anxiety	HADS, STAI	8	279/226	SMD=1.35, 95% CI−2.09 to−0.60, I^2=93%	+ P<0.00001		
						Reduction of Depression	BDI, CES-D	12	417/344	SMD=0.98, 95%CI: [−1.64 to−0.32], I^2=94%	+ P<0.0001		
						Reduction of stress	PSS	4	102/83	WMD=7.03, 95% CI: [12.11 to−1.95], I^2=98%	+ P<0.0001		
						Reduction of fatigue levels	BFI, MFSI, FACIF-F, FACT-B, VAS	14	463/419	SMD=−0.99 , 95%CI: [−1.56 to−0.43], I^2=93%	+ P<0.0001		
						Reduction of pain severity	BPI, VAS, SF36	5	156/132	SMD=-0.38, 95%CI: [−0.74	+ P=0.05		

Reference	Author (year)	Aim	Participants	Intervention	Comparator	Outcomes	Outcome measures	Nr of RCTs for outcome	Total nr of participants (intervention/control)	Effect size (95% CI), heterogenity	Significancy (P-value)	Overall conclusion	*Comment (by Marit Mentink)*
										to−0.02], I^2=58%			
						Reduction of sleep disturbance	PSQI, VAS	5	170/162	WMD=−0.99,95% CI: [−1.95 to−0.04], I^2=85%	+ P<0.0001		
[30]	Hu et al (2022)	To evaluate the clinical efficacy and safety of Traditional Medicine Preparations (TMPs) combined with chemotherapy for the treatment of Advanced pancreatic cancer (APC)	Patients diagnosed with unresectable (locally advanced and/or metastatic) or stage III–IV pancreatic cancer	TMP (extracts of herbs, patented herbal products, or self-prepared herbal decoctions) + chemotherapy	Chemotherapy	Quality of life	Continuous	9	302/298	SMD=0.81, 95% CI: [0.44 to 1.18, I^2=78%	+ P<0.0001	TMPs combined with chemotherapy significantly improved QoL. Moreover, TMPs reduced the adverse drug reactions (ADRs) during chemotherapy.	
							KPS	4	165/163	RR=1.44, 95% CI: [1.22 to 1.70], I^2=0%	+ P<0.0001		
						Gastrointestinal reaction	incidence	5	123/120	RR=0.33, 95% CI [0.12-0.90], I^2=0%	+ P=0.03		
						Nausea and vomiting	Incidence	5	216/215	RR=0.67, 95% CI [0.38-1.17], I^2=0%	- P=0.15		
						Hair loss	Incidence	3	165/165	RR=1.00, 95% CI [0.21-4.86],	- P=1.00		

Reference	Author (year)	Aim	Participants	Intervention	Comparator	Outcomes	Outcome measures	Nr of RCTs for outcome	Total nr of participants (intervention/control)	Effect size (95% CI), heterogenity	Significancy (P-value)	Overall conclusion	*Comment (by Marit Mentink)*
										I^2=0%			
[31]	Huang et al (2020)	To evaluate the effect of Traditional Chinese Medicine Injection (TCMJ) on cancer-related fatigue (CRF)	Patients with cancer-related fatigue, clear diagnosis, and unlimited cancer types	TCMJ (all types) and control group treatment	Chemotherapy	Clinical efficacy of CRF	RECIST	6	286/263	RR=1.24, 95% CI: [1.05–1.46], I^2=0%	+ P=0.01	TCMJ could improve fatigue status, and quality of life of CRF patients in a short period of time (≤4 weeks), but there was no statistical significance in the fatigue score	*TCMJ had advantages in improving fatigue of lung cancer and gastric cancer and life quality of lung cancer patients*
						Fatigue status	Piper fatigue scale (binary)	8	363/338	RR=1.44, 95% CI: [1.27–1.65], I^2=40%	+ P<0.00001		
						Fatigue score	Piper fatigue scale (continuous)	3	95/95	MD=−1.10, 95% CI: [−2.23–0.04], I^2=90%	- P=0.06		
						Quality of life	KPS	4	104/104	MD=8.34, 95% CI: [3.31–13.37], I^2=70%	+ P=0.001		
[32]	Jang et al (2020)	To assess the evidence of acupuncture for the treatment of cancer related fatigue (CRF)	Various types of cancer patients	Acupuncture with or without electrostimulation	Sham acupuncture	CRF	BFI	4	N/R	SMD=0.93, 95% CI: [-1.65,-0.20], I^2 not reported	+ P-value not reported	Acupuncture has therapeutic potential in management of CRF for cancer survivors	

Refer nce	Author (year)	Aim	Participants	Intervention	Comparat or	Outcomes	Outcome measures	Nr of RCTs for outco me	Total nr of participants (intervention/c ontrol)	Effect size (95% CI), heterogenit y	Significancy (P-value)	Overall conclusion	***Comment (by Marit Mentink)***
					Usual care			3	N/R	SMD=2.12, 95% CI: [-3.21, -1.04], I^2 not reported	+ P-value not reported		
[33]	Jang et al (2020)	To investigate the efficacy and safety of acupuncture in relation to the various symptoms induced by breast cancer treatment.	Women currently being treated for breast cancer	Acupuncture (manual, ear)	Sham or relaxation	Daily hot flash frequency	N/A	3	95/82	MD=0.3; 95%CI: [-0.84, 1.44]; I^2=47%	- P=0.61	There is currently insufficient evidence to conclude that acupuncture is of any benefit to patients suffering from symptoms caused by cancer treatment	
					Wait list	Night time hot flash frequency	N/A	2	49/47	MD=-0.83; 95%CI: [-1.94, 0.28]; I^2= 95%	- P=0.14		
						Menopausal symptoms	Kupperman index	3	62/64	MD=-2.61; 95%CI: [-7.35, 2.14]; I^2=99%	- P=0.28		
[34]	Jihong et al (2021)	To evaluate the effectiveness of yoga on cancer-related fatigue (CRF) in patients undergoing chemotherapy and/or	Adults undergoing chemotherapy and/or radiation therapy for cancer	Yoga	Routine care	Cancer related fatigue	BFI, CFS, FACIT-F, Piper fatigue scale, MFI, EORTC-QLQ-C30	11	664/629	SMD =–0.52, 95% CI: [–0.86, –0.18], I^2=88%	+ P=0.003	Yoga interventions had a positive effect in reducing CRF among patients undergoing chemotherapy and/or radiation	*Subgroup analysis demonstrated that yoga type, intervention strategy, weekly and total duration, and assessment instrument were significant factors that affected the reported*

Reference	Author (year)	Aim	Participants	Intervention	Comparator	Outcomes	Outcome measures	Nr of RCTs for outcome	Total nr of participants (intervention/control)	Effect size (95% CI), heterogenity	Significancy (P-value)	Overall conclusion	*Comment (by Marit Mentink)*
		radiation therapy										therapy, but the adherence to yoga was low	*effectiveness of yoga on CRF*
[35]	Jin et al (2020)	To determine the effectiveness and safety of acupuncture for chemotherapy-induced peripheral neuropathy	Patients who had malignant tumor received chemotherapy with peripheral neurotoxic drugs and had symptoms related to peripheral nerve injury	Acupuncture or electroacupuncture	Conventional care, sham acupuncture	Neurotoxicity	FACT-NTX	3	118/115	MD=−1.82, 95% CI: [−2.03 to −1.62]; I^2=99%	+ P<0.00001	We can see the positive effect of acupuncture in the analysis of FACT-NTX score. Result shows that acupuncture has a certain effect on improving pain. Heterogeneity of the results is high.	
						Pain	BPI	4	132/130	MD=−1.64, 95% CI: [−1.71 to −1.58]; I^2=99%	+ P<0.00001		
[36]	Jing et al (2018)	To review data from randomized controlled trials (RCTs) of auricular acupressure therapy for preventing constipation in leukemia patients undergoing	Patients diagnosed with leukemia who were over 18 years of age and undergoing chemotherapy	Auricular acupressure (+routine care)	Routine care	Constipation	Constipation Assessment scale (CAS)	3	116/116	MD =-1.51, 95% CI: [1.89, 1.14], I^2=0%	+ P<0.01	Only weak evidence supported the hypothesis that AA effectively prevented constipation in leukemia patients undergoing	

Refer nce	Author (year)	Aim	Participants	Intervention	Comparat or	Outcomes	Outcome measures	Nr of RCTs for outco me	Total nr of participants (intervention/c ontrol)	Effect size (95% CI), heterogenit y	Signific ancy (P-value)	Overall conclusion	*Comment (by Marit Mentink)*
		chemotherap y										chemothera py	
						Quality of life	PAC-QOL	2	86/86	MD=-1.28, 95% CI: [1.44, 1.13], I^2=0%	+ P<0.01		
[37]	Kanna n et al (2022)	To determine the efficacy of physical therapy interventions on quality of life (QoL) and pain severity in postmastecto my pain syndrome (PMPS)	Women with postmastec tomy pain syndrome (PMPS)	Acupuncture (elektro)	Wait list, usual care	Reduction in pain severity	BPI-SF, NPRS	2	24/31	SMD=−0.82 , 95%CI: [−1.36 to −0.29], I^2 not reported	+ P=0.003	The effect of acupuncture , myofascial release remains inconclusive	
				Myofascial release (Pilat approach, massage)	Placebo			2	32/33	SMD= −0.65, 95%CI: [−1.29 to −0.01], I^2 not reported	+ P=0.04		
[38]	Kuo et al (2021)	To explore the clinical effects of the Baduanjin Qigong exercise among cancer patients	Diagnosed with cancer	Baduanjin Qigong exercise	Routine care	Cancer related fatigue	BFI	5	186/184	OR=0.27; 95% CI: [0.17, 0.42], I^2=0%	+ P<0.000 01	The Baduanjin exercise had positive clinical effects on cancer patients. -is meta-analysis not only supported that the Baduanjin	

Refer nce	Author (year)	Aim	Participants	Intervention	Comparat or	Outcomes	Outcome measures	Nr of RCTs for outco me	Total nr of participants (intervention/c ontrol)	Effect size (95% CI), heterogenit y	Significancy (P-value)	Overall conclusion	*Comment (by Marit Mentink)*
												exercise can alleviate the degree of cancer-related fatigue in patients but also improved their quality of life and sleep quality	
						Quality of life	EORTC-QLQ-C30	2	96/94	MD=10.57 (95% CI [7.82, 13.32], I^2=92%	+ P<0.00001		
						Quality of life	FACT-B	3	117/110	MD=11.04, 95% CI: [9.56, 12.53], I^2=88%	+ P<0.00001		
						Sleep quality	PSQI (higher score indicates worsened sleep quality)	2	58/59	MD=-2.89, 95% CI: [−3.48, −2.30], I^2=0%	+ P<0.001		
[39]	Kwon et al (2021)	To evaluate the effectiveness and safety of herbal medicines (HMs) on cancer-related fatigue in patients with lung cancer	Lung cancer patients with CRF	Herbal medicine (oral) + conventional medicine	Placebo, no treatment, conventional treatment	Severity of cancer-related fatigue (CRF)	BFI global score	3	Total =220	MD=−1.47; 95% CI: [−1.64 to −1.29], I^2=98%	+ P<.00001	HM combined with conventional medicine significantly improved fatigue level, QOL, and ADL.	*Other fatigue scales (PFS, CFS) also yielded significant results for HMs*

Reference	Author (year)	Aim	Participants	Intervention	Comparator	Outcomes	Outcome measures	Nr of RCTs for outcome	Total nr of participants (intervention/control)	Effect size (95% CI), heterogenity	Significancy (P-value)	Overall conclusion	***Comment (by Marit Mentink)***
						Quality of life	QLQ-C30 global health status	2	Total=135	MD=8.05; 95% CI: [5.97, 10.14], I^2=35%	+ P<0.00001		
						Activities of daily life (ADL)	KPS	5	Total=328	MD=6.81; 95% CI: [4.34 to 9.28], I^2=96%	+ P<0.00001		
[40]	Li et al (2021)	To assess effect of acupuncture combined with three-step analgesic drugs to treat cancer pain	Cancer patients	Acupuncture combined with three-step analgesic drugs	Three-step analgesic treatment	Cancer pain	Relief response rate	18	679/672	RR=1.12, 95% CI: [1.08-1.17], I^2=19%	+ P<0.00001	For the treatment of cancer pain, acupuncture combined with three-step analgesic drugs is better than using only three-step analgesic drugs	
							NRS	7	282/281	SMD=−1.10, 95% CI: [−1.86, −0.35], I^2=96%	+ P=0.004		
							Burst pain rate	4	244/244	SMD=-1.38; 95% CI: [-2.44, 0.32], I^2=95%	+ P=0.01		
						Side effects	Incidence of nausea	8	N/R	RR=0.45, 95% CI: [0.38, 0.53], I^2=1%	+ P<0.00001	Compared with three-step analgesia alone, acupuncture combined	

Reference	Author (year)	Aim	Participants	Intervention	Comparator	Outcomes	Outcome measures	Nr of RCTs for outcome	Total nr of participants (intervention/control)	Effect size (95% CI), heterogenity	Significancy (P-value)	Overall conclusion	*Comment (by Marit Mentink)*
												with three-step analgesia for cancer pain reduced the rate of side effects	
							Incidence of vomiting	11	N/R	RR=0.56, 95% CI: [0.37, 0.86], I^2=0%	+ P=0.008		
							Incidence of constipation	11	N/R	RR=0.38, 95% CI: [0.29, 0.49], I^2=4%	+ P<0.00001		
							Incidence of dizziness	5	N/R	RR=0.53, 95% CI: [0.33, 0.86], I^2=10%	+ P=0.010		
[41]	Li et al (2021)	To evaluate the effect of acupuncture on treatment-related symptoms among breast cancer survivors	Breast cancer survivors	Verum acupuncture or electroacupuncture	Sham acupuncture, waitlist, usual care, another intervention	Pain intensity	N/R	3	N/R	SMD=-0.60, 95% CI: [-1.06 to -0.15], I^2=77%	+ P-value not reported	Acupuncture significantly reduces multiple treatment-related symptoms compared with the usual care or waitlist control group among breast cancer survivors	*No significant effects were found in subgroup analysis with sham acupuncture as control group*
					Sham acupuncture, waitlist,	Fatigue	N/R	4	N/R	SMD=-0.62, 95% CI: [-1.03 to -	+ P-value not		*No significant effects were found in subgroup analysis with sham*

Refer nce	Author (year)	Aim	Participants	Intervention	Comparat or	Outcomes	Outcome measures	Nr of RCTs for outco me	Total nr of participants (intervention/c ontrol)	Effect size (95% CI), heterogenit y	Signific ancy (P-value)	Overall conclusion	*Comment (by Marit Mentink)*
					usual care, another interventio n					0.20], I^2=68%	reporte d		*acupuncture as control group*
					Waitlist, usual care	Hot flash severity	N/R	2	N/R	SMD=-0.74, 95% CI: [-1.00 to 0.48], I^2=0%	+ P<0.000 01		
					Sham	Hot flash frequency	N/R	3	N/R	SMD=-0.29, 95% CI: [-0.83 to 0.24), I^2=72%	- P=0.28		
					Other interventio ns	Sleep disturbance	N/R	2	N/R	SMD=-0.19, 95% CI: [-0.98 to 0.61], I^2=73%	- P=0.65		
					Sham	Depression	N/R	2	N/R	SMD=-0.05, 95% CI: [-0.42 to 0.31], I^2=0%	- P=0.77		
[42]	Li et al (2019)	To evaluate the efficacy and safety of Chinese herbal medicine (CHM) for the treatment of depression or depressive symptoms in cancer patients	Cancer patients fulfilling the diagnostic criteria for depression or presented with depressive symptoms	Chinese Herbal Medicine (CHM)	No treatment	Depression symptom scores		7	286/272	SMD=-2.30 95% CI: [3.54, − 1.05], I^2=97%	+ P=0.000 3	The CHM intervention appears to alleviate depressive symptoms in cancer patients, either alone or combined with antidepress ants or psychologica l treatments	*Xiao Yao decoction and its modifications were the most frequently used*
						Depression treatment		4	167/149	RR=1.65, 95% CI: [1.19 to	+ P=0.003		

Reference	Author (year)	Aim	Participants	Intervention	Comparator	Outcomes	Outcome measures	Nr of RCTs for outcome	Total nr of participants (intervention/control)	Effect size (95% CI), heterogenity	Significancy (P-value)	Overall conclusion	*Comment (by Marit Mentink)*
						response rate				2.29]; I^2=33%			
						Treatment response rate - quality of life		2	49/53	RR=1.60, 95% CI: [1.17 to 2.18]; I^2=0%	+ P=0.003		
					Antidepressant	Depression symptom scores		7	268/268	SMD=-0.61, 95% CI: [-1.03 to –0.18]; I^2=82%	+ P<0.0001		
						Depression treatment response rate		6	227/227	RR=1.08, 95% CI: [0.93 to 1.26], I^2=18%	- P=0.31		
						Gastrointestinal disorders		6	238/238	RR=0.24, 95% CI: [0.08 to 0.69]; I^2=46%	+ P=0.008		
						Headache		5	197/197	RR=0.50, 95%CI: [0.21 to 1.19], I^2=0%	- P=0.12		
						Sleep disturbances		4	129/129	RR=0.41, 95% CI: [0.19 to 0.88]; I^2=0%	+ P=0.02		
						Dry mouth		4	137/137	RR=0.36, 95% CI: [0.08 to 1.62], I^2=63%	- P=0.18		
						Blurred vision		2	94/94	RR=0.08, 95% CI: [0.01 to 0.63]; I^2=0%	+ P=0.02		

Reference	Author (year)	Aim	Participants	Intervention	Comparator	Outcomes	Outcome measures	Nr of RCTs for outcome	Total nr of participants (intervention/control)	Effect size (95% CI), heterogenity	Significancy (P-value)	Overall conclusion	*Comment (by Marit Mentink)*
				CHM + psychological treatment	Psychological treatment	Depression treatment response rate		2	69/69	RR=1.70, 95% CI: [1.02 to 2.85], I^2=61%	+ P=0.04		
[43]	Li et al (2020)	To evaluate the efficacy of Chinese Herbal Medicine (CHM) as adjuvant therapy for reducing the chemotherapy-induced side-effects in the treatment of breast cancer	Patients with breast cancer toxicity grade 0-II	Chinese Herbal medicine (CHM)	Placebo or conventional western medicine	Chemotherapy induced vomiting and nausea (CINV)-grade 0-II	Various (frequency, grade, KPS, WHO criteria)	8	282/283	RR=1.27, 95% CI: [1.15 to 1.40, I^2=9%	+ P<0.00001	The adjunctive use of CHM with chemotherapy may reduce the chemotherapeutic agents-associated adverse events, including nausea and vomiting, diarrhea, alopecia	*CHM belonging to different TCM theory showed different magnitude of therapeutic effects*
			Patients with breast cancer toxicity grade III-IV			CINV grade III-IV		28	919/855	RR=0.39, 95% CI: [0.32 to 0.48], I^2=0%	+ P<0.00001		
						Diarrhea	WHO criteria, CTCAE 3.0	4	120/121	OR=0.30, 95% CI: [0.16 to 0.55], I^2=0%	+ P<0.0001		
						Constipation	CTCAE3.0V	2	54/54	RR=0.61, 95% CI: [0.32–1.17], I^2=0%	- P=0.14		
						Alopecia	KPS, QoL	2	128/129	RR=0.46, 95% CI: [0.23–0.91], I^2=0%	+ p=0.03		

Refer nce	Author (year)	Aim	Participants	Intervention	Comparat or	Outcomes	Outcome measures	Nr of RCTs for outco me	Total nr of participants (intervention/c ontrol)	Effect size (95% CI), heterogenit y	Significancy (P-value)	Overall conclusion	*Comment (by Marit Mentink)*
[44]	Li et al (2020)	To compare the efficacy and safety of traditional Chinese medicines (TCMs) combined with paclitaxel-based chemotherapy and paclitaxel-based chemotherapy alone for gastric cancer treatment	Gastric cancer patients	Traditional chinese medicines (TCM) +	Paclitaxel-based chemotherapy	Improvement of quality of life	KPS	4	122/124	RR=1.53; 95% CI: [1.19–1.96; I^2=0%	+ P<0.0001	Compared with paclitaxelbased chemotherapy alone, the combination of TCMs and paclitaxel-based chemotherapy may increase the TRR, improve quality of life, and reduce multiple chemotherapy-related side effects in gastric cancer patients	
						Nausea and vomiting	n/r	8	280/282	RR=0.50; 95% CI: [0.32–0.80], I^2=85%	+ P<0.01		
[45]	Li et al (2020)	To assess the effectiveness of music therapy on the quality of life, anxiety, depression and pain of	Population with a current cancer diagnosis	Music therapy + standard care	Standard care	Overall quality of life	Fact-G, EORTC QLQ-C30, HQoLI-R, QoL-CA, KPS	10	410/390	SMD=0.54, 95% CI: [0.40, 0.69], I^2=49%	+ p<0.00001	Music therapy can improve the overall quality of life of patients with	

Reference	Author (year)	Aim	Participants	Intervention	Comparator	Outcomes	Outcome measures	Nr of RCTs for outcome	Total nr of participants (intervention/control)	Effect size (95% CI), heterogenity	Significancy (P-value)	Overall conclusion	*Comment (by Marit Mentink)*
		patients with cancer										cancer, with an observed optimal intervention duration of 1–2 months. Meanwhile, anxiety, depression and pain are improved as well.	
						Anxiety	STAI-S, HADS, SAS	6	223/224	SMD=-1.51, 95% CI: [−2.27, 0.75], I^2=91%	+ p<0.00001		
						Depression	SDS, HAMS, BDI, CESE, HADS	6	277/278	SMD=−1.12, 95% CI: [−1.87, −0.38], I^2=94%	+ p<0.00001		
						Pain	VAS, NRS, VASP	5	190/191	SMD=−0.73, 95% CI: [−0.94, −0.52], I^2=0%	+ p<0.00001		
[46]	Li et al (2021)	To study the efficacy and safety of Chinese Medicine (CM) combined Western Medicine (WM) as an adjuvant treatment for reducing side effects	Postoperative breast cancer patients under treatment of endocrine therapy	Chinese Medicine (herbal formulae)+ endocrine therapy	Placebo CM + endocrine therapy	Quality of life	FACT-B	2	315/320	MD=0.73, 95% CI: [0.11–1.35], I^2=0%	+ P=0.02	The adjunctive use of CM reduced the endocrine therapy associated adverse events, perimenopausal symptoms, poor	

Reference	Author (year)	Aim	Participants	Intervention	Comparator	Outcomes	Outcome measures	Nr of RCTs for outcome	Total nr of participants (intervention/control)	Effect size (95% CI), heterogenity	Significancy (P-value)	Overall conclusion	*Comment (by Marit Mentink)*
		induced by endocrine therapy in breast cancer patients										quality of life, pain	
					Endocrine therapy	Menopausal-like symptoms	Kupperman scale	3	1132/1107	MD=-2.35, 95% CI: [−2.76 to −1.94], I^2=97%	+ P<0.00001		
						Improvement of performance status	KPS	4	108/108	MD=3.76, 95% CI: [1.64–5.88], I^2=38%	+ P=0.0005		
						Quality of life	FACT-B	4	462/447	MD=3.01, 95% CI: [1.00–5.02], I^2=97%	+ P=0.003		
						Pain status	VAS	3	90/90	MD=-2.35, 95% CI: [-3.40 to -1.30], I^2=81%	+ P<0.0001		
[47]	Li et al (2019)	To assess the effectiveness and safety on the method of activating blood and dredging collaterals in traditional Chinese medicine (TCM) for reducing CIPN	Adults diagnosed with cancer and received chemotherapy	Herbal medicines (with the function of activating blood and dredging collaterals)	No intervention, placebo, western medication,	Chemotherapy induced peripheral neuropathy (CIPN) grade 1-4	Incidence rate, Levi's grade, NCI-CTC, WHO grade, TCSS	15	571/522	OR=0.26, 95% CI: [0.20, 0.35], I^2=18.8%	+ P<0.00001	Herbs with the function of activating blood and dredging collaterals were found to potentially promote the curative effects	
[48]	Lin et al (2019)	To evaluate the effects of complementary and	Patients with cancer	Yoga	Usual care	Change in HRQOL	Various (FACT, FACIT, MDASI, SF36, EORTC)	7	282/237	MD=0.19, 95%CI: [-6.56 to 6.94], I^2=0%	- P=0.96	CIM may improve the HRQOL of	

Reference	Author (year)	Aim	Participants	Intervention	Comparator	Outcomes	Outcome measures	Nr of RCTs for outcome	Total nr of participants (intervention/control)	Effect size (95% CI), heterogenity	Significancy (P-value)	Overall conclusion	*Comment (by Marit Mentink)*
		integrative medicine (CIM) on the health-related quality of life (HRQOL) of cancer patients										cancer patients	
				Chinese Herbal medicine				4	144/143	MD=6.03, 95%CI: [-0.15 to 11.92], I^2=0%	+ P=0.04		
				Qigong				3	160/158	MD=3.01, 95%CI[-3.00 to 9.01]. I^2=0%	- P=0.33		
				Acupuncture (manual needle)				3	157/158	MD=0.80, 95%CI: [-9.36 to 10.95], I^2=0%	- P=0.88		
				Massage				2	85/84	MD=-5.00, 95%CI: [-39.01 to 29.01], I^2=0%	- P=0.77		
[49]	Lin et al (2022)	The effects of mindfulness-based stress reduction (MBSR) and mindfulness-based cognitive therapy (MBCT) on quality of life (QOL), pain, fatigue,	Various types of cancer patients	MBSR	Usual care, wait list, no treatment	QoL	Various (FACT-G, FACT-B, SF36, EORTC-QLQ-30)	7	454/446	SMD=0.96, 95% CI: [0.21–1.71], I^2=96%	+ P<0.00001	MBCT/MBSR is used to alleviate pain, fatigue, anxiety, and depression, and improve QOL in cancer patients	*Better effects found on short-term, in younger patients and early cancer stages*

Reference	Author (year)	Aim	Participants	Intervention	Comparator	Outcomes	Outcome measures	Nr of RCTs for outcome	Total nr of participants (intervention/control)	Effect size (95% CI), heterogenity	Significancy (P-value)	Overall conclusion	*Comment (by Marit Mentink)*
		anxiety, and depression in cancer patients											
						Anxiety	Various (HAM-A, HAD-A, GAD-7, CEC, STAI)	14	756/744	SMD=-0.55, 95%CI: [-0.93 to -0.17], I^2=92%	+ P=0.005		
						Depression	Various (HAD-D, POMS, PHQ-8)	12	667/646	SMD=-0.51, 95%CI [-0.74 to -0.28], I^2=73%	+ P<0.00001		
						Pain	Various (NRS, BPI)	4	241/246	SMD=-0.27, 95% CI [-0.44 to -0.09], I^2=11%	+ P=0.003		
						Fatigue	Various (POMS, MDASI)	8	432/429	SMD=-0.54, 95% CI [-0.84 to -0.23], I^2=76%	+ P<0.00001		
[50]	Lin et al (2022)	To meta-analyze the effectiveness of manual lymphatic drainage (MLD) in breast cancer-related lymphedema (BCRL) patients	Women prevented and/or treated for BCRL	MLD with or without control intervention	Active control, usual care, passive control	Pain	VAS, NRS, EQ-5D-5L	3	89/84	SMD=-0.72, 95% CI: [-1.34 to -0.09], I^2=74%	+ P=-0.02	Pain of BCRL patients undergoing MLD is significantly improved, while our findings do not support the use of MLD in improving quality of life	
						Quality of life	EORTC QLQ C30, NRS,	4	114/109	SMD=0.26, 95% CI: [-	- P=0.06		

Reference	Author (year)	Aim	Participants	Intervention	Comparator	Outcomes	Outcome measures	Nr of RCTs for outcome	Total nr of participants (intervention/control)	Effect size (95% CI), heterogenity	Significancy (P-value)	Overall conclusion	*Comment (by Marit Mentink)*
							Lymph-ICF, EQ-5D-5l			0.01 to 0.52], I^2=0%			
[51]	Liu et al (2021)	To systematically evaluate the intervention effect of mind-body exercise on cancer-related fatigue (CRF) in breast cancer patients	Adults diagnosed with breast cancer and CRF	Tai chi (2 studies, one Taiji Qigong)	N/R	Improvement in cancer related fatigue	Various (BFI, EORTC QLQ-C30, CFS, FACIT-F< FSI, PFS-R)	2	n/r	SMD=0.96 , 95%CI: [0.10, 1.82], I^2= 67%	+ P=0.08	Doing Tai Chi for ＞40 minutes each time with an exercise cycle of ≤6 weeks has a better effect on relieving CRF in breast cancer patients	
				Yoga	N/R			13	n/r	SMD=0.59 95% CI: [0.18, 0.99], I^2=88%	+ P=0.004		
[52]	Liu et al (2020)	To compare the therapeutic efficacy and safety of nonhormonal hot flash treatments for breast cancer survivors	Women who had been diagnosed with breast cancer and who were experiencing hot flashes	Acupuncture	Placebo/sham	Hot flash frequency	N/A	3	106/92	SMD=–2.42, 95% CI: [–6.93, 2.09], I^2=89%	- P=0.29	Acupuncture might be more effective in improving hot flashes for breast cancer survivors	*A pronounced placebo response was found during hot flash treatments*
[53]	Liu et al (2020)	To evaluate the current evidence for the effectiveness of tai chi in patients with breast cancer	Adult patients diagnosed with breast cancer who received active breast cancer treatment	Tai chi (also combined with qigong)	Conventional supportive interventions	Fatigue (3m after intervention)	FSI, CFS, BFI	2	85/89	MD=-0.46, 95% CI: [-1.09 to 0.17], I^2=0%	- P=0.15	Tai chi significantly relieves fatigue and QoL for breast cancer patients when used with	

Reference	Author (year)	Aim	Participants	Intervention	Comparator	Outcomes	Outcome measures	Nr of RCTs for outcome	Total nr of participants (intervention/control)	Effect size (95% CI), heterogenity	Significancy (P-value)	Overall conclusion	*Comment (by Marit Mentink)*
												conventional supportive care interventions	
						Sleeping quality (3m)	PSQI, AISI	2	76/82	MD=0.26, 95% CI: [-1.28 to 1.80], I^2=58%	- P=0.74		
						Quality of life (3m)	SF-36	2	472/480	SMD=0.32, 95% CI: [0.07 to 0.56], I^2=67%	+ P=0.01		
						Depression (3m)	IDS, SDS, BDI, CES-D	3	108/103	SMD=0.22 95% CI: [-0.05 to 0.49], I^2=0%	- p=0.12		
				Tai chi + conventional therapy	Conventional therapy	Fatigue (3m)	FSI, CFS, BFI	2	60/56	SMD=-0.91, 95% CI: [-1.30 to -0.53], I^2=0%	+ P<0.00001		
						Quality of life (3m)	WHOQoL-BREF	4	1194/1188	SMD=0.34, 95% CI: [0.26 to 0.43], I2=0%	+ P<0.00001		
[54]	Liu et al (2019)	To assess the clinical evidence for integrative herbal medicine therapy in the management of chemotherapy induced	Adults diagnosed with colorectal, colon or rectal cancer	Herbal medicine + chemotherapy	Placebo, conventional therapy, no intervention	Chemotherapy-induced peripheral neurotoxicity (all grades)	NCI-CTCAE	9	Total = 727	RR=0.74, 95% CI: [0.58, 0.94], I^2=13.5%	+ P value not reported	Integrative herbal therapy appeared to reduce CIPN and HFS in people receiving chemotherapy for CRC	*Grade III+IV were not statistically significant*

Reference	Author (year)	Aim	Participants	Intervention	Comparator	Outcomes	Outcome measures	Nr of RCTs for outcome	Total nr of participants (intervention/control)	Effect size (95% CI), heterogenity	Significancy (P-value)	Overall conclusion	*Comment (by Marit Mentink)*
		peripheral neuropathy (CIPN) and hand-foot syndrome (HFS) resulting from treatments for colorectal cancer											
						Hand-foot syndrome (all grades)	NCI-CTCAE	5	Total = 397	RR=0.93, 95% CI [0.55, 1.55], I^2=75.7%	- P-value not reported		
[55]	Liu et al (2021)	To clarify the clinical and placebo effects of acupuncture in treating Aromatase Inhibitors Arthralgia (AIA)	Patients with breast cancer (hormone receptor-positive) taking Ais for >1 month	Acupuncture (electro, auricular, body)	Sham, waitlist, drug	Pain-related interference	BPI	4	328/231	MD=−1.89, 95% CI: [−2.99, −0.79], I^2=91%	+ P=.008	Acupuncture is a safe and effective treatment for breast cancer patients with AIA	*There were no significant differences between the acupuncture group and sham-acupuncture group*
						Pain severity	BPI	3	143/168	MD=−1.57, 95% CI: [−2.46, −0.68], I^2=82%	+ P=.0006		
						Worst pain	BPI	3	152/186	MD=−2.31, 95% CI: [−3.15, −1.48], I^2=83%	+ P<.0001		
[56]	Lu (2021)	To assess benefits and negative effects	Adults with inoperable stage III-IV NSCLC	Chinese Herbal Medicine + Tyrosine kinase	Tyrosine kinase inhibitors (TKI), placebo	Performance status	KPS	2	137/133	MD=6.65; 95% CI: [5.81–7.49]; I^2=0%	+ p<0.00001	CHM reduces AE induced by EGFR-TKIs	

Refer nce	Author (year)	Aim	Participants	Intervention	Comparat or	Outcomes	Outcome measures	Nr of RCTs for outco me	Total nr of participants (intervention/c ontrol)	Effect size (95% CI), heterogenit y	Signific ancy (P-value)	Overall conclusion	*Comment (by Marit Mentink)*
		of Chinese Herbal Medicine as a combination therapy with first-generation EGFR-TKIs in advanced non small cell lung cancer (NSCLC) patients		inhibitors (TKI)									
						Incidence of diarrhea	n/a	8	466/445	RR=0.43, 95% CI: [0.30–0.60]; I^2=0%	+ p < 0.00001		
[57]	Luo et al (2020)	To evaluate the effect of TCC in breast cancer patients	Adult female patients who were diagnosed breast cancer	Tai chi chuan (TCC)	Non-exercise interventio n, usual care, blank control	Quality of life	WHOQOLBREF, FACT-B, MOSSF-36,	5	163/165	SMD=0.37, 95% CI: [0.15–0.59], I^2= 0%	+ p=0.001	TCC appears to be effective on some physical and psychologica l symptoms and improves the quality of life in patients with breast cancer	*12-25 weeks significant, 3-6 weeks not*
						Pain	n/r	4	169/168	SMD=0.30, 95% CI: [0.08–0.51], I^2=0%	+ p=0.007		
						Anxiety	n/r	2	120/116	MD=−4.25, 95% CI: [−5.87 to	+ p<0.000 01		

Reference	Author (year)	Aim	Participants	Intervention	Comparator	Outcomes	Outcome measures	Nr of RCTs for outcome	Total nr of participants (intervention/control)	Effect size (95% CI), heterogenity	Significancy (P-value)	Overall conclusion	*Comment (by Marit Mentink)*
										−2.63], I^2=80%			
						Fatigue	FSI	3	83/77	SMD=−1.11, 95% CI: [−1.53 to −0.69], I^2=30%	+ p<0.00001		
[58]	Ma (2019)	To investigate whether moxibustion can improve cancer-related fatigue	Adult cancer survivors (incl. active treatment)	Moxibustion	Conventional care, sham moxibustion	Fatigue	EORTC-QLQ-C30, CFS, PFS-R	10	Total=780	SMD=-1.17; 95% CI: [-1.44, -0.90], I^2=67%	+ P<0.00001	Moxibustion can improve cancer-related fatigue and most aspects of quality of life	
						QoL	EORTC-QLQ-C30,	9	Total=471	SMD=0.88, 95% CI: [0.54, 1.22], I^2=67%	+ P<0.00001		
[59]	Mai et al (2022)	To systematically evaluate the effect of acupressure on cancer pain	The participants were diagnosed with cancer, and tumors caused the pain	Acupressure + Standard care	Standard care	Pain remission rate	n/r	11	487/487	RR=1.20, 95%CI: [1.10, 1.30], I^2=61%	+ P<0.0001	Acupressure (including auricular acupressure) group seemed to be more effective than the control group for cancer pain	
						Pain intensity	n/r	22	989/989	SMD=-1.78, 95% CI: [-2.21, -1.35], I^2=94%	+ P<0.00001		
						Quality of life	n/r	8	297/294	SMD=0.62, 95%CI: [0.35,	+ P<0.00001		

Reference	Author (year)	Aim	Participants	Intervention	Comparator	Outcomes	Outcome measures	Nr of RCTs for outcome	Total nr of participants (intervention/control)	Effect size (95% CI), heterogenity	Significancy (P-value)	Overall conclusion	*Comment (by Marit Mentink)*
										0.89], I^2=61%			
						Sleep quality	PSQI and others	7		SMD=−1.94, 95%CI: [−2.96, −0.93], I^2=96%	+ P=0.0002		
[60]	Ni et al (2019)	To assess the effects of Tai Chi on quality of life (QOL) of cancer survivors	Adults with a diagnosis of cancer	Tai chi	Usual care or sham qigong	Physical functioning (QoL)	WHOQOL-BREF, FACT-B, SF-36	8	331/348	SMD=0.34, 95%CI: [0.09, 0.59]; I^2=59%	+ P=0.007	There is low-level evidence suggesting a positive effect of Tai Chi on physical and mental functioning and sleep quality	
						Psychological functioning (QoL)	WHOQOL-BREF, FACT-B, SF-36	8		SMD=0.60, 95%CI: [0.12, 1.08], I^2=89%	+ P=0.01		
						Social functioning (QoL)	WHOQOL-BREF, FACT-B, SF-36	7	292/303	SMD=0.26, 95%CI: [−0.25, 0.77]; I^2=89%	- P=0.32		
						Fatigue	BFI	3	115/119	SMD=−0.37, 95%CI: [−0.70, −0.04]; I^2= 80%	+ P=0.03		
						Sleep quality	PSQI, SRSS	3	106/112	SMD=−0.26, 95%CI: [−0.02, 0.53]; I^2=94%	+ P=0.07		

Refer nce	Author (year)	Aim	Participants	Intervention	Comparat or	Outcomes	Outcome measures	Nr of RCTs for outco me	Total nr of participants (intervention/c ontrol)	Effect size (95% CI), heterogenit y	Significancy (P-value)	Overall conclusion	*Comment (by Marit Mentink)*
[61]	Ni et al (2020)	To evaluate the preventive and therapeutic effect of acupuncture for radiation-induced xerostomia in cancer patients	Adult cancer patients with symptoms of dry mouth caused by radiotherapy or patients with cancer who will undergo radiotherapy	Acupuncture (body and ear)	Sham acupuncture	Xerostomia	Xerostomia Questionnaire	2	124/130	MD=−3.05, 95% CI: [−5.58 to −0.52], I^2=0%	+ P=0.02	Acupuncture is effective at improving xerostomia symptoms in cancer patients	
[62]	O'Neill et al (2020)	To determine the role of yoga interventions in improving cancer related fatigue and quality of life in women with breast cancer	Women with breast cancer	Yoga	Non-active	Fatigue	FSI, EORTC QLQ-C30, BFI, FACT-F	18	Total=1066	SMD=-0.30, 95% CI: [-0.51, −0.08], I^2=62%	+ P-value not reported	Yoga provides small to medium improvements in CRF and QOL compared to non-physical activity (PA), but not in comparison to other PA interventions	*Results suggesting that yoga has similar benefits as physical activity, but better than no activity at all*
						Quality of life	EORTC QLQ-C30, FAC, MOSF, LQLQ	10	Total=568	SMD=0.27, 95% CI: [0.46, 0.07], I^2=21%	+ P-value not reported		
					Active (exercise, health education)	Fatigue		6	n/r	SMD=−0.17 , 95% CI: [−0.50,	- P-value not		

Reference	Author (year)	Aim	Participants	Intervention	Comparator	Outcomes	Outcome measures	Nr of RCTs for outcome	Total nr of participants (intervention/control)	Effect size (95% CI), heterogenity	Significancy (P-value)	Overall conclusion	*Comment (by Marit Mentink)*
										0.17], I^2=55%	reported		
						Quality of life		3	Total=223	SMD=-0.04, 95% CI: [0.22, −0.31], I^2=0%	- P-value not reported		
[63]	Pinheiro da Silva et al (2019)	To investigate the effectiveness of manual therapy (MT) for chronic musculoskeletal pain (CMP) in the upper limbs and thorax of female breast cancer survivors and to investigate the changes in the quality of life and function	Women diagnosed with breast cancer who had completed treatment	Manual therapy (massage, myofascial release)	Active control, no intervention, usual care	Chronic musculoskeletal pain intensity	VAS, pain pressure threshold, GCI	5	127/112	SMD=-0.32 95% CI: [-0.57, -0.06], I^2=0%	+ P=0.02	Current evidence suggests that MT is considered effective for treating CMP in the upper limbs and thorax of female breast cancer survivors	
						Quality of life	SF-356, EORTC QLQ BR23, FACT-B.	3	71/85	SMD=-0.14, 95% CI: [-0.46, 0.17], I^2=0%	- P=0.38		
[64]	Qi et al (2021)	To critically evaluate the effect of music interventions on cancer-related fatigue (CRF) in cancer patients	Adults diagnosed with any type of cancer	Music therapy	Usual care, active control	Fatigue	BFI, VAS, POMS, MFSI-SF	8	235/232	SMD=−0.88, 95% CI: [−1.49, −0.26], I^2=89%	+ P=0.005	Music interventions can be considered as an alternative therapy for relieving fatigue in cancer	*Listening to prerecorded music has no effect compared to live music*

Refer nce	Author (year)	Aim	Participants	Intervention	Comparat or	Outcomes	Outcome measures	Nr of RCTs for outco me	Total nr of participants (intervention/c ontrol)	Effect size (95% CI), heterogenit y	Signific ancy (P-value)	Overall conclusion	*Comment (by Marit Mentink)*
												patients who are undergoing active treatment or have completed treatment.	
[65]	Qiao et al (2022)	To analyze the efficacy of manual lymphatic drainage (MLD) for breast cancer related postmastectomy lymphedema (BCRL)	Patients with BCRL	Manual lymphatic drainage	Usual care	Pain	n/r	2	67/66	SMD=−0.09 ; 95% CI: [−0.43 to 0.25], I^2=0%	- P=.61	Manual lymphatic drainage may not have a positive effect on reducing pain in the upper limbs.	
[66]	Schell et al (2019)	To assess the effects of mindfulness-based stress reduction (MBSR) in women diagnosed with breast cancer.	Adult women with a confirmed diagnosis of breast cancer	Mindfulness based stress reduction (MBSR) + anticancer therapy	Anticancer therapy	Fatigue	POMS< FSI, SCL-90-R	5	348/345	SMD=−0.50 , 95% CI: [−0.86 to −0.14], I^2=77.18%	+ P=0.01	MBSR may improve quality of life slightly at the end of the intervention but may result in little to no difference later on.	*Effects on anxiety, depression, sleep quality up to 6 months, but no effect for QoL and fatigue.*
						Anxiety	GAD-7, POMS, SCL-90-R, STAI	6	383/366	SMD=−0.29 , 95% CI: [−0.50 to −0.0], I^2=40.2%	+ P=0.01		

Reference	Author (year)	Aim	Participants	Intervention	Comparator	Outcomes	Outcome measures	Nr of RCTs for outcome	Total nr of participants (intervention/control)	Effect size (95% CI), heterogenity	Significancy (P-value)	Overall conclusion	*Comment (by Marit Mentink)*
						Depression	CES-D, PHQ-8, POMS, SCL-90-R	6	384/361	SMD=−0.54, 95% CI: [−0.86 to −0.22]; I^2=72.85%	+ P=0.0010		
						Sleep quality	PSQI, SCL-90-R, ISI	4	241/234	SMD=−0.38, 95% CI: [−0.79 to 0.04], I^2=72.44%	+ P=0.01		
[67]	She et al (2021)	To evaluate the efficacy and safety of Chinese medicinal formulas (CMFs)	Intermediate-advanced primary liver cancer	CMFs + TACE	TACE treatment	Quality of life	KPS efficient rate	11	436/428	RR=1.42; 95%CI: [1.31, 1.55], I^2=0%	+ P< 0.00001	CMFs have the advantage of reducing certain serious adverse events	
						Nausea and vomiting	n/r	7	n/r	RR=0.71, 95% CI [0.57-0.89], I^2=0%	+ P=0.02		
						Inappetence	n/r	3	n/r	RR=0.66, 95% CI [0.47-0.91], I^2=0%	+ P=0.01		
[68]	Shi et al (2021)	To evaluate the clinical efficacy of Chinese medicine combined with neoadjuvant treatment of breast cancer	Breast cancer	Traditional Chinese Medicine (oral) + neoadjuvant chemotherapy	Chemotherapy	Performance status score efficiency	n/r	6	231/231	OR=2.61, 95% CI: [1.49-4.58], I^2=0%	+ P=0.0008	TCM combined with neoadjuvant chemotherapy to treat breast cancer has obvious advantages over chemotherapy alone in terms of the	

Reference	Author (year)	Aim	Participants	Intervention	Comparator	Outcomes	Outcome measures	Nr of RCTs for outcome	Total nr of participants (intervention/control)	Effect size (95% CI), heterogenity	Significancy (P-value)	Overall conclusion	*Comment (by Marit Mentink)*
												performance status score and the incidence of gastrointestinal adverse reactions after chemotherapy	
						Incidence of adverse reactions in digestive system after chemotherapy	n/r	9	317/312	OR=0.04, 95% CI: [–0.01 to 0.09], I^2=0%	+ P=0.09		
[69]	Sinha et al (2021)	To evaluate the effect of the combined practice of PMR and GI on stress, anxiety, depression and mood	Patients with breast cancer	Relaxation + guided imagery	n/r	Stress		5	173/171	SMD=–0.38; 95% CI: [–0.59, –0.16]; I^2=8%	+ P < 0.001	The intervention was effective for stress and anxiety. It positively improved the quality of life but saw no significant improvement in chemotherapy-related adverse effects	*Not significant in radiotherapy subgroup*
						Anxiety		5	173/171	SMD=–0.30; 95% CI, [–0.51, –0.09]; I^2=24%	+ P=0.006		*Not significant in radiotherapy subgroup*

Reference	Author (year)	Aim	Participants	Intervention	Comparator	Outcomes	Outcome measures	Nr of RCTs for outcome	Total nr of participants (intervention/control)	Effect size (95% CI), heterogenity	Significancy (P-value)	Overall conclusion	*Comment (by Marit Mentink)*
						Depression		5	173/190	SMD=−0.37; 95% CI, [−0.81, 0.07]; I^2=76%	+ P=0.10		*Not significant in radiotherapy/chemotherapy subgroups*
						Quality of life		2	78/78	SMD=0.60; 95% CI: [0.28, 0.92]; I^2=0%	+ P=0.0002		
						Nausea and vomiting		2	62/63	SMD=−0.50; 95% CI: [−1.07, 0.06]; I^2=60%	+ P=0.08		*Contains only patients receiving chemotherapy*
[70]	Song et al (2018)	To evaluate current evidence and estimate the pooled effects of Tai Chi on cancer related fatigue (CRF)	Patients with a confirmed diagnosis of cancer and symptoms of CRF	Tai chi	Active control, usual care	Fatigue (post-intervention)	BFI, FACIT-F, MFSI-SF, FSI	6	179/194	SMD=−0.54; 95% CI: [−0.75, −0.33]; I^2=32%	+ P<0.0001	Tai Chi for more than 8 weeks has short-term ameliorative effects on CRF	
						Fatigue (after 3m)		2	87/132	SMD=0.23; 95% CI: [-0.61, 1.08], I^2=89%	- P=0.59		
[71]	Suh et al (2021)	To evaluate the efficacy and effectiveness of a mindfulness-based stress reduction (MBSR) program in improving sleep in cancer survivors	Adult cancer survivors	MBSR	Usual care	Sleep quality	PSQI, ISI, MOS	9	398/389	SMD=−0.29, 95% CI: [−0.55 to − 0.04], I^2=58%	+ P=0.02	MBSR helps cancer survivors improve sleep quality more than usual care	

Reference	Author (year)	Aim	Participants	Intervention	Comparator	Outcomes	Outcome measures	Nr of RCTs for outcome	Total nr of participants (intervention/control)	Effect size (95% CI), heterogenity	Significancy (P-value)	Overall conclusion	*Comment (by Marit Mentink)*
					Active control		ISI	3	169/150	SMD=0.10, 95 % CI: [-0.13 to 0.32], I^2=90%	- P=0.40		
							PSQI	3	169/150	SMD=0.27, 95% CI: [0.04 to 0.50], I^2=96%	+ P=0.02		
[72]	Tan et al (2021)	To identify the research evidence on acupoint stimulation (AS) for cancer-related fatigue (CRF) management	Adult cancer patients with fatigue	Acupuncture	Sham	Fatigue	Piper Fatigue Scale, MFI, BFI, MFI-20, Tang Fatigue Rating Scale, FSS, VAS	2	Total = 123	SMD=-0.29, 95% CI: [-0.65 to 0.07], I^2<50%	- P=0.11	Acupoint stimulation was more effective in alleviating CRF than standard treatment/care	
				Acupressure	Sham			2	Total = 100	SMD=-0.26, 95% CI: [-0.66 to 0.14], I^2<50%	- P=0.20		
[73]	Tang et al (2019)	To determine whether walking is more effective than yoga at improving sleep disturbance in cancer patients	Adult patient with any cancer diagnosis	Yoga	Wait list, usual care	Sleep disturbance	PSQI	13	Total = 1227	SMD=-0.26, 95% CI: [-0.42 to -0.10], I^2=36.81%	+ P=0.002	Walking is more effective than yoga in improving sleep disturbance in cancer patients	
[74]	Wang et al (2020)	To evaluate the effectiveness of combining Chinese	Advanced non-small cell lung cancer (stage III-IV)	CHM + platinum-based chemotherapy	Platinum-based chemotherapy alone	Quality of life	KPS	4	138/139	SMD=1.47; 95% CI: [0.30–2.64]; I^2= 94.41%	+ P=0.014	Higher QOL associated with CHM use as an adjuvant to	

Reference	Author (year)	Aim	Participants	Intervention	Comparator	Outcomes	Outcome measures	Nr of RCTs for outcome	Total nr of participants (intervention/control)	Effect size (95% CI), heterogenity	Significancy (P-value)	Overall conclusion	*Comment (by Marit Mentink)*
		Herbal Medicine (CHM) treatment and platinum-based therapy										chemotherapy	
[75]	Wang et al (2021)	To evaluate the efficacy of Qigong Exercise for alleviating fatigue	Patients with primary disease (including cancer), separately analyzed	Qigong	Active, sham	Fatigue	Various (e.g. MFI-20, CFS, FACT-G, FSS)	4	176/208	SMD=-0.75; 95% CI: [-1.37 to -0.13]; I^2=86%	+ P=0.02	Qigong exercise may be beneficial for improving fatigue in patients diagnosed with various diseases	
[76]	Wang et al (2019)	To evaluate the efficacy and safety of Chinese Herbal Medicine in the treatment of Ovarian cancer after reduction surgery and adjuvant chemotherapy	Patients were confirmed with diagnosis of ovarian cancer	CHM + WM	Western medicine	Performance status	KPS	9	418/422	MD=3.90, 95% CI: [1.22–6.58], I^2=91%	+ P=0.004	CHM combined with WM is effective and safe as a treatment for OvC patients after reduction surgery and adjuvant chemotherapy	
						Quality of life	n/r	3	180/176	MD=2.55, 95% CI: [0.01–5.10], I^2=79%	- P=0.05		
						Nausea and vomiting	n/r	7	160/160	RR=0.72, 95% CI: [0.55 to 0.94], I^2=77%	+ P=0.02		

Reference	Author (year)	Aim	Participants	Intervention	Comparator	Outcomes	Outcome measures	Nr of RCTs for outcome	Total nr of participants (intervention/control)	Effect size (95% CI), heterogenity	Significancy (P-value)	Overall conclusion	*Comment (by Marit Mentink)*
						Diarrhea	n/r	2	35/35	RR=0.87, 95% CI: [0.55 to 1.36], I^2=0%	- P=0.53		
						Neuropathy	n/r	3	76/73	RR=1.11, 95% CI: [0.81–1.50], I^2=0%	- P=0.52		
[77]	Wang et al (2020)	To examine the effectiveness and safety of yoga of women with sleep problems	Adult women with sleep problems, including breast cancer patients (separate analysis)	Yoga	Wait list, active control, usual care	Sleep quality	PSQI	7	304/301	SMD=−0.15; 95% CI: [−0.31 to 0.01]; I^2=0%	- P=0.07	No evidence for the effect of yoga compared with the control group in improving sleep quality for women with breast cancer using the PSQI	
[78]	Wang et al (2018)	To critically assess the effectiveness and safety of acupuncture for treating hot flashes (HFs) among breast cancer (BC) patients	Breast cancer patients with hot flashes	Acupuncture (traditional)	Sham	Hot flash frequency (during treatment)	n/a	3	249/206	MD=–1.78, 95% CI: [–3.42-–0.14], I^2=69%	+ P=0.03	Acupuncture still appeared to be an efficacious therapeutic strategy	*Posttreatment and follow up effects of acupuncture on hot flash frequency were not significant*
[79]	Wang et al (2021)	To assess the effect of oral Chinese medicine (OCM) combined with Western	Adult patients with cancer pain	OCM + WM	Western medicine with or without placebo	QoL	KPS, QoL score	7	382/383	MD=8.41, 95% CI: [4.46, 12.36], I^2=92%	+ P<0.0001	Compared with the WM, OCM combined with WM could significantly relieve the	

Reference	Author (year)	Aim	Participants	Intervention	Comparator	Outcomes	Outcome measures	Nr of RCTs for outcome	Total nr of participants (intervention/control)	Effect size (95% CI), heterogenity	Significancy (P-value)	Overall conclusion	*Comment (by Marit Mentink)*
		medicine (WM)										cancer pain, improve and the QOL	
						Pain relief rate	NRS	14	544/543	RR=1.43, 95% CI: [1.32, 1.56], I^2=0%	+ P<0.00001		
[80]	Wang et al (2021)	To synthesize evidence of the efficacy and safety of auricular acupressure for cancer patients with sleep disturbance	Adult cancer patients with diagnosis of sleep disturbance	Auricular acupressure	Routine care, sham acupuncture, no intervention	Sleep quality	PSQI	6	241/244	MD=−3.88, 95% CI: [−5.24, −2.53], I^2=91%	+ P<0.00001	Auricular acupressure can significantly improve the sleep quality of cancer patients with sleep disturbance	
[81]	Wu et al (2019)	The efficacy of traditional Chinese medicine preparation combined with chemotherapy	Patients diagnosed with cancer	CHM + chemotherapy	Chemotherapy	Gastrointestinal reaction (anorexia, nausea, vomiting)	n/r	2	352/316	RR=1.12, 95% CI (0.75 to 1.69), I^2=68%	- P=0.57	No obvious advantage in gastrointestinal reactions	
[82]	Xie et al (2020)	To critically evaluate the effects of mindfulness-based stress reduction (MBSR) on cancer-related fatigue (CRF)	Adult cancer patients	MBSR with or without usual care	Usual care or without intervention	Fatigue	PFS-R, CFS, FSI, MDASI, POMS	14	1502/1506	SMD=−0.89, 95%CI: [-1.19, −0.59], I^2=93%	+ P <.001	MBSR is effective for CRF management and can be recommended as a beneficial complementary therapy for CRF patients	*8 weeks of MBSR, supervised by experts, had a large effect on CRF*

Reference	Author (year)	Aim	Participants	Intervention	Comparator	Outcomes	Outcome measures	Nr of RCTs for outcome	Total nr of participants (intervention/control)	Effect size (95% CI), heterogenity	Significancy (P-value)	Overall conclusion	*Comment (by Marit Mentink)*
[83]	Xu et al (2021)	To evaluate the effectiveness and safety of acupuncture for quality of life of patients with defecation dysfunction (DD) after sphincter preserving surgery for rectal cancer	Adults with with DD after sphincter preserving surgery for rectal cancer	Acupuncture (manual, electro)	Active control (biofeedback, exercise)	Urination	QLQ-CR29	2	100/100	MD=−0.39 95% CI: [−0.46 to −0.32]; I^2=34%	+ P=0.0007	Acupuncture or electropuncture may be effective and safe for DD, but the quality of included studies was very low.	
						Abdominal pain		2	100/100	MD=−0.71, 95% CI: [−0.89 to −0.54]; I^2= 9%	+ P<0.0001		
						Stool		2	100/100	MD=−0.49; 95% CI: [−0.77 to −0.20]; I^2=57%	+ P=0.003		
						Defecation		2	100/100	MD=−0.59; 95% CI: [−0.85 to −0.33]; I^2=51%	+ P<0.0001		
						Sexual function		2	100/100	MD=0.93; 95% CI: [0.48 to 1.38]; I^2=90%	+ P<0.00001		
						Self-feelings		2	100/100	MD=1.04; 95% CI: [0.36 to 1.73]; I^2=94%	+ P<0.0001		

Refer nce	Author (year)	Aim	Participants	Intervention	Comparat or	Outcomes	Outcome measures	Nr of RCTs for outco me	Total nr of participants (intervention/c ontrol)	Effect size (95% CI), heterogenit y	Signific ancy (P-value)	Overall conclusion	*Comment (by Marit Mentink)*
[84]	Xunlin et al (2019)	To systematically summarize and synthesize the best available evidence concerning the effectiveness of mindfulness-based interventions on anxiety, depression, quality of life, fatigue, stress, posttraumatic growth, and mindfulness among adult cancer patients and survivors	Adult cancer patients or survivors	MBSR		Anxiety	STAI, HADS, POMS, HAM-A	9	Total=1053	SMD=− 0.30 95% CI: [−0.45, −0.14), I^2=34%	+ P=0.000 3	Mindfulness -based intervention s can be used as an adjuvant therapy for the managemen t of cancer-related symptoms among cancer patients and survivors	
[85]	Yang et al (2020)	To systematically evaluate the efficacy of Traditional Chinese Medicine (TCM) combined with chemotherap y and provide evidence-based	Patients with non-small cell lung cancer	TCM + chemotherap y	Chemothe rapy	Quality of life	n/r	5	219/219	OR=2.79, 95% CI: [1.87, 4.16], I^2=42%	+ P<0.000 01	TCM combined with chemothera py can improve clinical efficacy and KPS score, as well as improve patients' QOL and reduce ADRs	

Reference	Author (year)	Aim	Participants	Intervention	Comparator	Outcomes	Outcome measures	Nr of RCTs for outcome	Total nr of participants (intervention/control)	Effect size (95% CI), heterogenity	Significancy (P-value)	Overall conclusion	*Comment (by Marit Mentink)*
		evidence for the treatment of non-small cell lung cancer										caused by chemotherapy drugs	
						Performance status	KPS	4	157/152	OR=2.88, 95% CI: [1.79, 4.62], I^2=54%	+ P<0.00001		
						Nausea and vomiting	n/r	9	426/418	OR=0.16, 95% CI: [0.11, 0.22], I^2=34%	+ P<0.00001		
						Diarrhea	n/r	3	168/159	OR=0.21, 95% CI: [0.12, 0.37], I^2=0%	+ P<0.00001		
[86]	Yang et al (2021)	To systematically evaluate the effectiveness of five-element music therapy on anxiety, depression, quality of life (QoL), sleep quality and Karnofsky performance score (KPS) in cancer patients	Participants were diagnosed with any type of cancer	Five element music therapy	Usual care, active control	Depression	SDS, HAMD	14	659/649	SMD =−1.11, 95% CI: [1.41 to −0.82], I^2=83%	+ P<0.00001	Five-element music therapy had a positive effect on depression, QoL, sleep quality, and KPS in cancer patients, while did not show a positive effect on anxiety	
						Quality of life	HRQOL, EORTC-QLQ-C30	7	343/311	SMD=1.41, 95% CI: [0.58 to 2.23], I^2=95%	+ P=0.0008		

Refer nce	Author (year)	Aim	Participants	Intervention	Comparat or	Outcomes	Outcome measures	Nr of RCTs for outco me	Total nr of participants (intervention/c ontrol)	Effect size (95% CI), heterogenit y	Significancy (P-value)	Overall conclusion	*Comment (by Marit Mentink)*
						Sleep quality	PSQI	4	168/168	MD=−1.73, 95% CI: [2.34 to − 1.12], I^2=68%	+ P<0.00001		
						Performance status	KPS	3	135/103	MD=4.75, 95% CI: [2.31 to 7.18], I^2=37%	+ P=0.0001		
						Anxiety	SAS, HAMA	8	350/350	SMD =−0.60, 95% CI: [1.47 to 0.27], I^2=96%	- P=0.17		
[87]	Yangoz et al (2019)	To synthesize the effect of music intervention on patients with cancer-related pain in randomized controlled trials	Adults with cancer-related pain	Music intervention (passive listening)	Routine care, bed rest, music therapy	Pain	VAS, ONS, ESAS	6	Total = 593	Hedge's g=0.55, 95% CI: [0.19–0.92], I^2=76.83%	+ P=.003	Music intervention can have a positive effect on the management of cancer pain	
[88]	Yao et al (2022)	To assess whether moxibustion is effective and safe for gastrointestinal adverse effects	Patients with malignant tumours	Moxibustion (with or without western medicine)	Sham moxibustion, no intervention, Western medicine	Nausea and vomiting	n/r	24	1146/1131	RR=0.70, 95% CI: [0.61–0.79], I^2=70%	+ P<0.00001	Moderate- to very-low-quality evidence suggests that moxibustion may be safely used as an adjuvant treatment after	

Reference	Author (year)	Aim	Participants	Intervention	Comparator	Outcomes	Outcome measures	Nr of RCTs for outcome	Total nr of participants (intervention/control)	Effect size (95% CI), heterogenity	Significancy (P-value)	Overall conclusion	*Comment (by Marit Mentink)*
												chemotherapy	
						Diarrhoea	n/r	7	286/283	RR=0.56, 95% CI: [0.38–0.82], I^2=0%	+ P=0.003		
						Constipation	n/r	7	299/299	RR=0.59, 95% CI: [0.44–0.78], I^2=0%	+ P=0.0002		
						Abdominal distension	n/r	7	315/314	RR=0.60, 95% CI: [0.46–0.78], I^2=0%	+ P=0.0001		
						Inappetence	n/r	2	111/111	RR=0.69, 95% CI: [0.40–1.22], I^2=90%	- P=0.20		
						Abdominal pain	n/r	2	102/101	RR=0.69, 95% CI: [0.40–1.22], I^2=0%	- P=0.19		
						Performance status	KPS	6	178/173	MD=7.53, 95% CI: [3.42–11.64], I^2=89%	+ P=0.0003		
						Quality of life	EORTC-QLQ-C30	2	56/55	MD=8.88, 95% CI: [0.96–16.80], I^2=75%	+ P=0.03		
[89]	Yi et al (2021)	To assess the effects of yoga on health-related quality, physical	Women patients who were currently undergoing chemother	Yoga (includes meditation, breathing techniques, relaxation)	Usual care, active control (stretching, education)	Fatigue	BFI, MFI, FACT, PSQI	4	216/226	SMD=−0.62; 95% (CI): [−1.17 to −0.07], I^2=0%	+ P=0.03	Yoga may benefit to reduce fatigue, depression and anxiety, improve	*Only post-intervention effect, no medium- and long-term effects in fatigue, sleep disturbance*

Reference	Author (year)	Aim	Participants	Intervention	Comparator	Outcomes	Outcome measures	Nr of RCTs for outcome	Total nr of participants (intervention/control)	Effect size (95% CI), heterogenity	Significancy (P-value)	Overall conclusion	*Comment (by Marit Mentink)*
		health and psychological health in breast cancer patients undergoing chemotherapy	apy for breast cancer									sleep disturbance, and improve QoL in breast cancer patients receiving chemotherapy in the short-term	
						Sleep disturbance	PSQI	2	168/180	SMD=−0.34; 95% CI: [−0.55 to −0.12], I^2=0%	+ P=0.002		
						Depression	BDI,II, HADS , FACT-B, CES-D	6	236/210	SMD=−0.50; 95% CI: [−0.70 to −0.31], I^2=84%	+ P<0.00001		
						Anxiety	STAI, HADS, SDS	5	218/194	SMD=−0.50; 95% CI: [−0.70 to −0.31], I^2=46%	+ P<0.00001		
						Health related quality of life	EORtC-QLQ-C30	3	108/108	SMD=0.72; 95% CI: [−0.12 to 1.56], I^2=29%	+ P=0.09		
[90]	Yin et al (2020)	To evaluate clinical trial evidence of the effects of Qigong practice on self-reported fatigue among	Clinically diagnosed cancer patients or cancer survivors	Qigong	Conventional exercise	Fatigue	FACIT-F, MDASI, CRFS, FSI, BFI	13	Total = 1154	Mean effect size=0.46, 95% CI: [0.15 to 0.78], I^2=81.4%	+ P<0.0039	Qigong practice may have small-to-moderate efficacy for management of cancer-	*Significant effects of qigong on fatigue were not found when compared with Western exercise or no treatment control*

Reference	Author (year)	Aim	Participants	Intervention	Comparator	Outcomes	Outcome measures	Nr of RCTs for outcome	Total nr of participants (intervention/control)	Effect size (95% CI), heterogenity	Significancy (P-value)	Overall conclusion	*Comment (by Marit Mentink)*
		cancer patients or survivors										related fatigue	
[91]	Yoon et al (2021)	To evaluate the efficacy and safety of traditional herbal medicine (THM) for improving sleep quality in patients with cancer	Adults cancer patients with insomnia	THM	Hypnotics	Sleep quality	PSQI	6	209/207	MD=-2.25, 95% CI: [−3.46 to −1.05], I^2=84%	+ P=0.0002	THM may be an effective therapeutic option for insomnia in patients with cancer	*The most frequently used herbs were Ziziphus jujuba Mill*
					Placebo			2	81/81	MD=-2.56, 95% CI: [−3.81 to −1.31], I^2=91%	+ P<0.0001		
[92]	Yuanqing et al (2020)	To determine the efficacy of acupuncture on the management of hormone therapy–related side effects in breast cancer patients	Patients diagnosed with breast cancer actively undergoing breast cancer treatment and/or hormone therapy	Acupuncture, electroacupuncture, thermoacupuncture	Sham needles, wait list, progressive relaxation, routine care	Hot flashes	GCS, HFCS	7	Total = 578	SMD=−0.28; 95% CI: [−0.45 to −0.11]; I^2=47%	+ P=.00	Acupuncture is a moderately appropriate alternative therapy for hormone therapy–related side effects in breast cancer patients	*Evidence for outcome indicators of symptom management were downgraded by the GRADE system for inconsistency, indirectness, and imprecision in the included RCTs*
						Fatigue	BFI, BPI-SF	4	Total = 177	SMD=−1.19; 95% CI: [−2.25 to 0.12], I^2=86.7%	+ P=.02		
						Pain	VAS, BPI	5	Total = 319	SMD=−1.05; 95% CI: [−1.89 to	+ P=.01		

Reference	Author (year)	Aim	Participants	Intervention	Comparator	Outcomes	Outcome measures	Nr of RCTs for outcome	Total nr of participants (intervention/control)	Effect size (95% CI), heterogenity	Significancy (P-value)	Overall conclusion	*Comment (by Marit Mentink)*
										−0.21]; I^2=93%			
						Stiffness	WOMAC	5	Total = 316	SMD=−0.06; 95% CI: [-1.15 to −0.05]; I^2=85.7%	+ P=.03		
						Gastrointestinal symptoms	n/r	5	Total = 282	SMD=-0.09; 95% CI: [-0.32 to 0.15]; I^2=0%	- P=.47		
						Menopausal symptoms	Kupperman index	3	Total = 157	SMD=−0.36; 95% CI: [-1.08 to 0.37]; I^2=0.01%	- P=.34		
						Physical well-being	HAQ-DI, WOMAC, BPI, PPT	8	Total = 576	SMD=0.08; 95% CI: [-0.44 to 0.60]; I^2=86.5%	- P=.76		
						Social well-being	HADS, FACT-B	3	Total = 176	SMD=0.08; 95% CI: [-0.21 to 0.38]; I^2=0%	- P=.58		
						Emotional well-being	HADS, PSS, SCL, MS	3	Total = 176	SMD=−0.12; 95% CI: [-0.59 to 0.34]; I^2=53.8%	- P=.61		
[93]	Zeng et al (2022)	To explore the effect of hypnosis in breast cancer surgery	Adult female breast cancer patients	Hypnosis before general anesthesia	Usual care	Anxiety (preoperative)	n/r	6	499/480	MD=−2.79, 95% CI: [−3.93, −1.65], I^2=96%	+ P<0.00001	The application of hypnosis before general anesthesia for breast cancer surgery can reduce the	

Refer nce	Author (year)	Aim	Participants	Intervention	Comparat or	Outcomes	Outcome measures	Nr of RCTs for outco me	Total nr of participants (intervention/c ontrol)	Effect size (95% CI), heterogenit y	Signific ancy (P-value)	Overall conclusion	*Comment (by Marit Mentink)*
												degree of anxiety of patients, also reducing postoperativ e pain	
						Pain (post operative)	VAS	7	480/462	MD=−1.25, 95% CI: [−1.64, −0.86], I^2=93%	+ P<0.000 01		
						Nausea and vomiting (postoperati ve)		4	390/384	OR=0.68, 95%CI: [0.22, 2.07], I^2=71%	- P=0.49		
[94]	Zeng et al (2020)	To evaluate the comparative effects of all known nonpharmaco logical interventions for cancer-related cognitive impairment	Adult cancer patients	Yoga	Usual care, wait list	Cognitive impairment	BCPT, MDASI	2	Total = 514	MD=0.29, 95%CI: [-4.48, 5.07], I^2 not reported	- P-value not reporte d	No conclusion for yoga specifically	
[95]	Zhang et al (2018)	To assess the effects of moxibustion for alleviating side effects associated with chemotherap y, radiotherapy or both in	Participants with malignant disease receiving chemother apy, radiotherap y or both	Moxibustion + conventional medicine	Conventio nal medicine	Nausea and vomiting	WHO grade 3 to 4	7	425/376	RR=0.43 95%CI [0.25 to 0.74], I2=19%	+ P=0.002 1	Limited, low-certainty evidence suggests that moxibustion treatment may help to reduce gastrointesti nal	

Refer nce	Author (year)	Aim	Participants	Intervention	Comparat or	Outcomes	Outcome measures	Nr of RCTs for outco me	Total nr of participants (intervention/c ontrol)	Effect size (95% CI), heterogenit y	Significancy (P-value)	Overall conclusion	*Comment (by Marit Mentink)*
		people with cancer										toxicities of chemotherapy or radiotherapy, improving QoL in people with cancer	
						Performance status	KPS	4	127/125	MD=7.21, 95% CI [5.74 to 8.68], I2=0%	+ P<0.00001		
						QoL	EORTC QLQ C30	3	67/67	MD=8.85, 95% CI [4.25 to 13.46]	+ P=0.00016		
[96]	Zhang et al (2018)	To comprehensively aggregate and evaluate the available evidence on the efficacy and safety of the combination treatment with CHM and epidermal growth factor receptor tyrosine kinase inhibitors (EGFR-TKIs) in NSCLC patients	Adult patients with advances non-small cell lung cancer (NSCLC)	CHM (oral, intravenous, emulsion) + EFGR-TKI	EFGR-TKI (gefinitib, erlotinib, icotinib)	Cutaneous toxicity	WHO grade III or IV	20	Total = 1260	RR=0.52; 95% CI: [0.34 – 0.80]; I^2=0%	+ P=0.003	The combination treatment of CHM and EGFR-TKI may reduce severe toxicities and improve performance status in advanced NSCLC patients, compared with EGFR-TKIs alone	
						Diarrhea	WHO grade III or IV	15	Total = 956	RR=0.44; 95% CI:	+ P=0.003		

Reference	Author (year)	Aim	Participants	Intervention	Comparator	Outcomes	Outcome measures	Nr of RCTs for outcome	Total nr of participants (intervention/control)	Effect size (95% CI), heterogenity	Significancy (P-value)	Overall conclusion	*Comment (by Marit Mentink)*
										[0.25–0.76]; I^2=0%			
						Nausea and vomiting	WHO grade III or IV	3	Total = 253	RR=0.24; 95% CI: [0.06–0.92]; I^2=0%	+ P=0.037		
						Performance status	KPS	28	Total = 1764	RR=1.33; 95% CI: [1.26–1.41]; I^2=28.1%	+ P<0.0001		
[97]	Zhang et al (2021)	To evaluate the effects of acupuncture in women with breast cancer (BC), focusing on patient reported outcomes (PROs)	Adult females diagnosed with BC	Acupuncture (electro, manual)	Sham, no intervention, usual care	QoL	QLQ-C30,	2	Total = 154	MD=10.09, 95% CI: [7.26, 12.92], I^2=not reported	+ P<0.0001	Acupuncture might improve BC treatment-related symptoms measured with PROs including QoL, pain, fatigue, hot flashes, sleep disturbance and anxiety	*Still significant effect of acupuncture on QoL at extended follow up*
						Physical well being	FACT-G/B	2	Total = 345	MD=4.40, [1.49, 7.30], I^2=not reported	+ P=0.003		
						Social well being	FACT-G/B	2	Total = 345	MD=1.02 [0.01, 2.04	- P=0.05		
						Emotional well-being	FACT-G/B	2	Total = 345	MD=2.03 [1.11, 2.95], I^2=not reported	+ P<0.0001		
						Functional well-being	FACT-G/B	2	Total = 345	MD=3.49 [2.36, 4.62], I^2=not reported	+ P<0.0001		

Refer nce	Author (year)	Aim	Participants	Intervention	Comparat or	Outcomes	Outcome measures	Nr of RCTs for outco me	Total nr of participants (intervention/c ontrol)	Effect size (95% CI), heterogenit y	Significancy (P-value)	Overall conclusion	*Comment (by Marit Mentink)*
						Peripheral neuropathy	FACT-NTX	2	Total = 103	MD=4.40 [-1.58, 10.37], I^2=not reported	- P=0.15		
						Cognitive function	FACT-COG	2	Total = 93	MD=1.95 [-0.69, 4.60], I^2=not reported	- P=0.15		
						Pain severity	BPI-SF	4	307/201	SMD=-1.28, 95%CI: [-2.17 to -0.39], I^2=77%	+ P=0.005		
							VAS	3	80/80	SMD=-0.83, 95%CI: [-1.16 to -0.51], I^2=0%	+ P<0.00001		
						Hot flash score		2	175/195	MD=-4.08, 95% CI: [-7.98, -0.17], I^2=75%	+ P= 0.04		
						Hot flash frequency		4	133/120	MD=-0.47, 95% CI: [-1.56, 0.62], I^2=20%	- P=0.40		
						Fatigue	BFI, MFI-GF, PFS, QLQ-C30	6	295/295	SMD=-0.39, 95%CI: [-0.55 to -0.22], I^2=0%	+ P<0.00001		
						Sleep disturbance	PSQI, QLQ-C30	4	161/161	SMD=-0.50, 95%CI: [-0.71 to -0.28], I^2=0%	+ P<0.00001		
						Anxiety	HADS, NRS	2	80/81	SMD=-0.37, 95%CI: [-0.68 to -0.05], I^2=0%	+ P=0.02		

Reference	Author (year)	Aim	Participants	Intervention	Comparator	Outcomes	Outcome measures	Nr of RCTs for outcome	Total nr of participants (intervention/control)	Effect size (95% CI), heterogenity	Significancy (P-value)	Overall conclusion	*Comment (by Marit Mentink)*
						Depression	HADS, CESD, Beck	3	104/105	SMD=-0.17, 95%CI: [-0.52 to 0.19], I^2=40%	- P=0.36		
[98]	Zhang et al (2018)	To critically evaluate the effect of acupuncture on cancer-related fatigue (CRF)	Adult cancer patients	Acupuncture (electro and manual)	Sham acupuncture, usual care	Fatigue	BFI, MFI, ESAS, FACIT-F	5	496/353	SMD =−1.30, 95%CI: [−1.82, − 0.78], I^2=not reported	+ P<0.01	Acupuncture is effective for CRF management and should be recommended as a beneficial alternative therapy for CRF patients,	*Particular effects for breast cancer patients*
[99]	Zhao et al (2020)	To assess the effectiveness and safety of traditional Chinese medical therapy for cancer-related fatigue.	People with a clinical diagnosis of CRF	Chinese herbal medicine		Fatigue improvement rate	FACIT-F, BFI	10	356/349	SMD=1.02; 95% CI: [0.60-1.45], I^2=86%	+ P<0.00001	Chinese medical therapy seems to be effective and safe in the treatment of cancer-related fatigue	*CRF measured with dichotomous data also significant effect*
[100]	Zhu et al (2021)	To evaluate the effects of Physical Therapies (PTs) on improvement in psychosomatic symptoms and quality of	Diagnosis of breast cancer and receiving adjuvant therapy for aromatase inhibitors (Ais)	Acupuncture (traditional)	Sham acupuncture	Worst pain	BPI, WOMAC	4	n/r	SMD=−0.81, 95% CI: [−1.51, −0.11], I^2=83.5%	+ P-value not repoted	Based on moderate-level evidence, acupuncture was associated with significant	

Reference	Author (year)	Aim	Participants	Intervention	Comparator	Outcomes	Outcome measures	Nr of RCTs for outcome	Total nr of participants (intervention/control)	Effect size (95% CI), heterogenity	Significancy (P-value)	Overall conclusion	***Comment (by Marit Mentink)***
		life (QOL) in breast cancer patients										reductions in pain intensity in breast cancer patients treated with AIs. In psychosomatic symptoms, acupuncture and exercise training did not result in significant improvements.	
					Sham acupuncture, wait list	Pain severity	BPI	2	n/r	WMD=−1.18, 95% CI: [−1.91, -0.45], I^2=71.5%	- P-value not reported		
					Sham acupuncture, wait list	Pain interference	BPI	3	n/r	WMD=−1.07, 95% CI: [−1.69, -0.45], I^2=45.9%	- P-value not reported		
					Sham acupuncture	Anxiety	HADS	2	n/r	WMD=−0.21, 95% CI: [−3.44, 3.03], I^2=79.8%	P-value not reported		
					Sham acupuncture	Sleep disturbance	PSQI	2	n/r	WMD=0.98, 95%CI: [−0.57, 2.53], I^2=0%	P-value not reported		

N/A: not applicable, N/R: not reported

References

1. Armer, J.S. and S.K. Lutgendorf, *The impact of yoga on fatigue in cancer survivorship: a meta-analysis.* JNCI cancer spectrum, 2020. **4**(2): p. pkz098.
2. Bai, X., et al., *Effects of integrated Chinese traditional medicine and conventional western medicine on the quality of life of breast cancer patients: a systematic review and meta-analysis.* Evidence-Based Complementary and Alternative Medicine, 2022. **2022**.
3. Bro, M.L., et al., *Kind of blue: A systematic review and meta-analysis of music interventions in cancer treatment.* Psycho-oncology, 2018. **27**(2): p. 386-400.
4. Cai, Q., et al., *Tai Chi for anxiety and depression symptoms in cancer, stroke, heart failure, and chronic obstructive pulmonary disease: A systematic review and meta-analysis.* Complementary Therapies in Clinical Practice, 2022. **46**: p. 101510.
5. Chan, Y.-T., et al., *Systematic Review with Meta-Analysis: Effectiveness and Safety of Acupuncture as Adjuvant Therapy for Side Effects Management in Drug Therapy-Receiving Breast Cancer Patients.* Evidence-Based Complementary and Alternative Medicine, 2021. **2021**.
6. Chang, Y.-C., et al., *Short-term effects of randomized mindfulness-based intervention in female breast cancer survivors: a systematic review and meta-analysis.* Cancer Nursing, 2021. **44**(6): p. E703-E714.
7. Chen, C.-Y., X.-X. Lin, and X. Wang, *Efficacy of non-invasive auricular acupressure for treating constipation in leukemia patients undergoing chemotherapy: a systematic review.* Complementary Medicine Research, 2018. **25**(6): p. 406-412.
8. Chen, S., et al., *Efficacy and safety of TCM combined with chemotherapy for SCLC: a systematic review and meta-analysis.* Journal of Cancer Research and Clinical Oncology, 2020. **146**: p. 2913-2935.
9. Chen, L., et al., *Efficacy of auricular acupressure in prevention and treatment of chemotherapy-induced nausea and vomiting in patients with cancer: a systematic review and meta-analysis.* Evidence-Based Complementary and Alternative Medicine, 2021. **2021**: p. 1-11.
10. Chen, Y., et al., *Efficacy of Herbal Medicines Intervention for Colorectal Cancer Patients With Chemotherapy-Induced Gastrointestinal Toxicity—a Systematic Review and Meta-Analysis.* Frontiers in Oncology, 2021. **11**: p. 629132.
11. Chen, Z.-Q., et al., *Chinese herbal medicine for epidermal growth factor receptor inhibitor-induced skin rash in patients with malignancy: An updated meta-analysis of 23 randomized controlled trials.* Complementary Therapies in Medicine, 2019. **47**: p. 102167.
12. Chien, T.-J., et al., *The efficacy of acupuncture in chemotherapy-induced peripheral neuropathy: systematic review and meta-analysis.* Integrative cancer therapies, 2019. **18**: p. 1534735419886662.
13. Chien, T.-J., et al., *The maintenance effect of acupuncture on breast cancer-related menopause symptoms: a systematic review.* Climacteric, 2020. **23**(2): p. 130-139.
14. Coutiño-Escamilla, L., et al., *Non-pharmacological therapies for depressive symptoms in breast cancer patients: Systematic review and meta-analysis of randomized clinical trials.* The Breast, 2019. **44**: p. 135-143.
15. Dai, L., et al., *Acupuncture and derived therapies for pain in palliative cancer management: systematic review and meta-analysis based on single-arm and controlled trials.* Journal of Palliative Medicine, 2021. **24**(7): p. 1078-1099.
16. Danon, N., et al., *Are mind–body therapies effective for relieving cancer-related pain in adults? A systematic review and meta-analysis.* Psycho-Oncology, 2022. **31**(3): p. 345-371.

17. Deng, B. and W. Sun, *Herbal medicine for hand–foot syndrome induced by fluoropyrimidines: A systematic review and meta-analysis.* Phytotherapy Research, 2018. **32**(7): p. 1211-1228.
18. Dong, B., et al., *Yoga has a solid effect on cancer-related fatigue in patients with breast cancer: a meta-analysis.* Breast cancer research and treatment, 2019. **177**: p. 5-16.
19. Dong, B., et al., *Wrist-ankle acupuncture has a positive effect on cancer pain: a meta-analysis.* BMC complementary medicine and therapies, 2021. **21**(1): p. 1-10.
20. El-Hashimi, D. and K.M. Gorey, *Yoga-specific enhancement of quality of life among women with breast cancer: systematic review and exploratory meta-analysis of randomized controlled trials.* Journal of evidence-based integrative medicine, 2019. **24**: p. 2515690X19828325.
21. Gao, Y., et al., *Effects of Acupuncture and Moxibustion on Breast Cancer-Related Lymphedema: A Systematic Review and Meta-Analysis of Randomized Controlled Trials.* Integrative Cancer Therapies, 2021. **20**: p. 15347354211044107.
22. Gonzalez, M., et al., *Yoga for depression and anxiety symptoms in people with cancer: a systematic review and meta-analysis.* Psycho-Oncology, 2021. **30**(8): p. 1196-1208.
23. Haussmann, A., et al., *Meta-analysis of randomized controlled trials on yoga, psychosocial, and mindfulness-based interventions for cancer-related fatigue: what intervention characteristics are related to higher efficacy?* Cancers, 2022. **14**(8): p. 2016.
24. Jin, H., et al., *Traditional herbal medicine combined with first-line platinum-based chemotherapy for advanced non-small-cell lung cancer: A PRISMA-compliant systematic review and meta-analysis.* Medicine, 2021. **100**(37).
25. He, Y., et al., *Clinical evidence for association of acupuncture and acupressure with improved cancer pain: a systematic review and meta-analysis.* JAMA oncology, 2020. **6**(2): p. 271-278.
26. He, J., et al., *Mindfulness ased stress reduction interventions for cancer related fatigue: a meta-analysis and systematic review.* Journal of the National Medical Association, 2020. **112**(4): p. 387-394.
27. Hou, X.-B. and D.-D. Chen, *Effectiveness and safety of acupuncture on cancer pain: a meta-analysis.* TMR Integrative Medicine, 2020. **4**: p. e20018.
28. Hsieh, S.-H., et al., *The effect of acupressure on relieving cancer-related fatigue: a systematic review and meta-analysis of randomized controlled trials.* Cancer Nursing, 2021. **44**(6): p. E578-E588.
29. Hsueh, E.-J., et al., *Effects of yoga on improving quality of life in patients with breast cancer: a meta-analysis of randomized controlled trials.* Breast Cancer, 2021. **28**: p. 264-276.
30. Hu, J., et al., *Clinical efficacy and safety of traditional medicine preparations combined with chemotherapy for advanced pancreatic cancer: A systematic review and meta-analysis.* Frontiers in Oncology, 2022. **12**.
31. Huang, Z., et al., *Effect of traditional Chinese medicine injection on cancer-related fatigue: A meta-analysis based on existing evidence.* Evidence-Based Complementary and Alternative Medicine, 2020. **2020**.
32. Jang, A., et al., *The effects of acupuncture on cancer-related fatigue: updated systematic review and meta-analysis.* Integrative cancer therapies, 2020. **19**: p. 1534735420949679.
33. Jang, S., et al., *Acupuncture as an adjuvant therapy for management of treatment-related symptoms in breast cancer patients: systematic review and meta-analysis (PRISMA-compliant).* Medicine, 2020. **99**(50).

34. Song, J., *The effectiveness of yoga on cancer-related fatigue: a systematic review and meta-analysis.* Number 2/March 2021, 2021. **48**(2): p. 207-228.
35. Jin, Y., et al., *Efficacy and safety of acupuncture against chemotherapy-induced peripheral neuropathy: a systematic review and meta-analysis.* Evidence-Based Complementary and Alternative Medicine, 2020. **2020**.
36. Jing, X., et al., *Auricular acupressure is an alternative in treating constipation in leukemia patients undergoing chemotherapy: A systematic review and meta-analysis.* Complementary Therapies in Clinical Practice, 2018. **31**: p. 282-289.
37. Kannan, P., et al., *Efficacy of physical therapy interventions on quality of life and upper quadrant pain severity in women with post-mastectomy pain syndrome: a systematic review and meta-analysis.* Quality of Life Research, 2022. **31**(4): p. 951-973.
38. Kuo, C.-C., et al., *Clinical effects of Baduanjin Qigong exercise on cancer patients: a systematic review and meta-analysis on randomized controlled trials.* Evidence-Based Complementary and Alternative Medicine, 2021. **2021**: p. 1-10.
39. Kwon, C.Y., et al., *Effectiveness and safety of herbal medicine for cancer-related fatigue in lung cancer survivors: A systematic review and meta-analysis.* Phytotherapy Research, 2021. **35**(2): p. 751-770.
40. Li, D.-h., et al., *Acupuncture combined with three-step analgesic drug therapy for treatment of cancer pain: a systematic review and meta-analysis of randomised clinical trials.* Evidence-based Complementary and Alternative Medicine, 2021. **2021**: p. 1-12.
41. Li, H., et al., *Acupuncture improves multiple treatment-related symptoms in breast cancer survivors: A systematic review and meta-analysis.* The Journal of Alternative and Complementary Medicine, 2021. **27**(12): p. 1084-1097.
42. Li, M., et al., *Twelve Chinese herbal preparations for the treatment of depression or depressive symptoms in cancer patients: a systematic review and meta-analysis of randomized controlled trials.* BMC complementary and alternative medicine, 2019. **19**(1): p. 1-16.
43. Li, S., et al., *Chinese herbal medicine for reducing chemotherapy-associated side-effects in breast cancer patients: a systematic review and meta-analysis.* Frontiers in Oncology, 2020. **10**: p. 599073.
44. Li, Y., et al., *Meta-analysis of paclitaxel-based chemotherapy combined with traditional Chinese medicines for gastric cancer treatment.* Frontiers in Pharmacology, 2020. **11**: p. 132.
45. Li, Y., et al., *The effectiveness of music therapy for patients with cancer: A systematic review and meta-analysis.* Journal of Advanced Nursing, 2020. **76**(5): p. 1111-1123.
46. Li, L., et al., *Evidence on efficacy and safety of Chinese medicines combined western medicines treatment for breast cancer with endocrine therapy.* Frontiers in Oncology, 2021. **11**: p. 661925.
47. Li, Z., et al., *The method of activating blood and dredging collaterals for reducing chemotherapy-induced peripheral neuropathy: a systematic review and meta-analysis.* Evidence-Based Complementary and Alternative Medicine, 2019. **2019**.
48. Lin, W.-F., et al., *Efficacy of complementary and integrative medicine on health-related quality of life in cancer patients: a systematic review and meta-analysis.* Cancer management and research, 2019. **11**: p. 6663.
49. Lin, L.-Y., et al., *Effects of mindfulness-based therapy for cancer patients: A systematic review and meta-analysis.* Journal of Clinical Psychology in Medical Settings, 2022. **29**(2): p. 432-445.
50. Lin, Y., et al., *Manual lymphatic drainage for breast cancer-related lymphedema: a systematic review and meta-analysis of randomized controlled trials.* Clinical Breast Cancer, 2022.

51. Liu, C., et al., *A meta-analysis: intervention effect of mind-body exercise on relieving cancer-related fatigue in breast cancer patients.* Evidence-Based Complementary and Alternative Medicine, 2021. **2021**.
52. Liu, J., et al., *Nonhormonal hot flash management for breast cancer survivors: a systematic review and network meta-analysis.* Evidence-Based Complementary and Alternative Medicine, 2020. **2020**.
53. Liu, L., et al., *The effectiveness of tai chi in breast cancer patients: A systematic review and meta-analysis.* Complementary Therapies in Clinical Practice, 2020. **38**: p. 101078.
54. Liu, Y., et al., *Integrative herbal medicine for chemotherapy-induced peripheral neuropathy and hand-foot syndrome in colorectal cancer: a systematic review and meta-analysis.* Integrative cancer therapies, 2019. **18**: p. 1534735418817833.
55. Liu, X., et al., *Acupuncture for arthralgia induced by aromatase inhibitors in patients with breast cancer: a systematic review and meta-analysis.* Integrative Cancer Therapies, 2021. **20**: p. 1534735420980811.
56. Lu, Y., et al., *Chinese herbal medicine combined with first-generation EGFR-TKIs in treatment of advanced non-small cell lung cancer with EGFR sensitizing mutation: a systematic review and meta-analysis.* Frontiers in Pharmacology, 2021. **12**: p. 698371.
57. Luo, X.-C., et al., *Effect of Tai Chi Chuan in breast cancer patients: a systematic review and meta-analysis.* Frontiers in Oncology, 2020. **10**: p. 607.
58. Ma, H.-L., et al., *The effectiveness of moxibustion for cancer-related fatigue: An updated systematic review and meta-analysis.* European Journal of Integrative Medicine, 2019. **30**: p. 100960.
59. Mai, Q., et al., *Effects of acupressure on cancer-related pain management: A systematic review and meta-analysis of randomized controlled trials.* European Journal of Integrative Medicine, 2022: p. 102120.
60. Ni, X., et al., *The effects of Tai Chi on quality of life of cancer survivors: a systematic review and meta-analysis.* Supportive Care in Cancer, 2019. **27**: p. 3701-3716.
61. Ni, X., et al., *Acupuncture for radiation-induced xerostomia in cancer patients: a systematic review and meta-analysis.* Integrative Cancer Therapies, 2020. **19**: p. 1534735420980825.
62. O'Neill, M., et al., *The effect of yoga interventions on cancer-related fatigue and quality of life for women with breast cancer: a systematic review and meta-analysis of randomized controlled trials.* Integrative cancer therapies, 2020. **19**: p. 1534735420959882.
63. da Silva, F.P., et al., *Manual therapy as treatment for chronic musculoskeletal pain in female breast cancer survivors: a systematic review and meta-analysis.* Journal of Manipulative and Physiological Therapeutics, 2019. **42**(7): p. 503-513.
64. Qi, Y., et al., *Music interventions can alleviate cancer-related fatigue: a metaanalysis.* Supportive Care in Cancer, 2021. **29**: p. 3461-3470.
65. Qiao, J., et al., *Effect of Manual Lymphatic Drainage on Breast Cancer–Related Postmastectomy Lymphedema: A Meta-analysis of Randomized Controlled Trials.* Cancer Nursing, 2023. **46**(2): p. 159-166.
66. Schell, L.K., et al., *Mindfulness-based stress reduction for women diagnosed with breast cancer.* Cochrane Database of Systematic Reviews, 2019(3).
67. She, Y., et al., *the therapeutic principle of combined strengthening Qi and eliminating pathogens in treating middle-advanced primary liver cancer: a systematic review and meta-analysis.* Frontiers in Pharmacology, 2021: p. 2783.
68. Shi, G., et al., *A systematic review and meta-analysis of traditional Chinese medicine with chemotherapy in breast cancer.* Gland Surgery, 2021. **10**(5): p. 1744.

69. Sinha, M.K., et al., *Progressive muscle relaxation and guided imagery in breast cancer: a systematic review and meta-analysis of randomised controlled trials.* Indian Journal of Palliative Care, 2021. **27**(2): p. 336.
70. Song, S., et al., *Ameliorative effects of Tai Chi on cancer-related fatigue: a meta-analysis of randomized controlled trials.* Supportive Care in Cancer, 2018. **26**: p. 2091-2102.
71. Suh, H.-W., et al., *The mindfulness-based stress reduction program for improving sleep quality in cancer survivors: a systematic review and meta-analysis.* Complementary Therapies in Medicine, 2021. **57**: p. 102667.
72. Tan, J.-Y.B., et al., *Acupoint stimulation for cancer-related fatigue: A quantitative synthesis of randomised controlled trials.* Complementary Therapies in Clinical Practice, 2021. **45**: p. 101490.
73. Tang, M.-F., et al., *Walking is more effective than yoga at reducing sleep disturbance in cancer patients: A systematic review and meta-analysis of randomized controlled trials.* Sleep medicine reviews, 2019. **47**: p. 1-8.
74. Wang, L.-C., et al., *Systematic review and meta-analysis of Chinese herbal medicine as adjuvant treatment in advanced non-small cell lung cancer patients.* Complementary Therapies in Medicine, 2020. **52**: p. 102472.
75. Wang, R., et al., *Efficacy of qigong exercise for treatment of fatigue: a systematic review and meta-analysis.* Frontiers in medicine, 2021. **8**: p. 684058.
76. Wang, R., et al., *Efficacy and safety of Chinese herbal medicine on ovarian cancer after reduction surgery and adjuvant chemotherapy: a systematic review and meta-analysis.* Frontiers in Oncology, 2019. **9**: p. 730.
77. Wang, W.-L., et al., *The effect of yoga on sleep quality and insomnia in women with sleep problems: a systematic review and meta-analysis.* BMC psychiatry, 2020. **20**: p. 1-19.
78. Wang, X.-P., et al., *Acupuncture for the relief of hot flashes in breast cancer patients: A systematic review and meta-analysis of randomized controlled trials and observational studies.* Journal of cancer research and therapeutics, 2018. **14**(Suppl 3): p. S600-S608.
79. Wang, Y.-h., J.-y. Chang, and L. Feng, *Effect of oral Chinese medicine combined with Western medicine on cancer pain: a meta-analysis.* Chinese journal of integrative medicine, 2021. **27**: p. 713-720.
80. Wang, Y., et al., *Auricular acupressure therapy for patients with cancer with sleep disturbance: a systematic review and meta-analysis.* Evidence-Based Complementary and Alternative Medicine, 2021. **2021**.
81. Wu, J., et al., *Traditional Chinese medicine preparation combined therapy may improve chemotherapy efficacy: a systematic review and meta-analysis.* Evidence-Based Complementary and Alternative Medicine, 2019. **2019**.
82. Xie, C., et al., *Mindfulness-based stress reduction can alleviate cancer-related fatigue: a meta-analysis.* Journal of psychosomatic research, 2020. **130**: p. 109916.
83. Xu, G., et al., *Acupuncture for Quality of Life of Patients with Defecation Dysfunction after Sphincter Preserving Surgery for Rectal Cancer: A Systematic Review.* Evidence-Based Complementary and Alternative Medicine, 2021. **2021**.
84. Xunlin, N., Y. Lau, and P. Klainin-Yobas, *The effectiveness of mindfulness-based interventions among cancer patients and survivors: a systematic review and meta-analysis.* Supportive Care in Cancer, 2020. **28**: p. 1563-1578.

85. Yang, J., et al., *Efficacy of traditional Chinese Medicine combined with chemotherapy in patients with non-small cell lung cancer (NSCLC): a meta-analysis of randomized clinical trials.* Supportive Care in Cancer, 2020. **28**: p. 3571-3579.
86. Yang, T., et al., *Effectiveness of five-element music therapy in cancer patients: A systematic review and meta-analysis.* Complementary Therapies in Clinical Practice, 2021. **44**: p. 101416.
87. Yangöz, Ş.T. and Z. Özer, *The effect of music intervention on patients with cancer-related pain: A systematic review and meta-analysis of randomized controlled trials.* Journal of Advanced Nursing, 2019. **75**(12): p. 3362-3373.
88. Yao, Z., et al., *Moxibustion for alleviating chemotherapy-induced gastrointestinal adverse effects: a systematic review of randomized controlled trials.* Complementary Therapies in Clinical Practice, 2022: p. 101527.
89. Yi, L.-J., et al., *Effects of yoga on health-related quality, physical health and psychological health in women with breast cancer receiving chemotherapy: a systematic review and meta-analysis.* Ann Palliat Med, 2021. **10**(2): p. 1961-1975.
90. Yin, J., L. Tang, and R.K. Dishman, *The efficacy of Qigong practice for cancer-related fatigue: A systematic review and meta-analysis of randomized controlled trials.* Mental Health and Physical Activity, 2020. **19**: p. 100347.
91. Yoon, J.-H., et al., *Traditional herbal medicine for insomnia in patients with cancer: a systematic review and meta-analysis.* Frontiers in Pharmacology, 2021. **12**: p. 753140.
92. Yuanqing, P., et al., *Acupuncture for Hormone Therapy–Related Side Effects in Breast Cancer Patients: A GRADE-Assessed Systematic Review and Updated Meta-Analysis.* Integrative cancer therapies, 2020. **19**: p. 1534735420940394.
93. Zeng, J., et al., *Effect of hypnosis before general anesthesia on postoperative outcomes in patients undergoing minor surgery for breast cancer: a systematic review and meta-analysis.* Gland Surgery, 2022. **11**(3): p. 588.
94. Zeng, Y., et al., *Nonpharmacological interventions for cancer-related cognitive impairment in adult cancer patients: a network meta-analysis.* International journal of nursing studies, 2020. **104**: p. 103514.
95. Zhang, H.W., et al., *Moxibustion for alleviating side effects of chemotherapy or radiotherapy in people with cancer.* Cochrane Database of Systematic Reviews, 2018(11).
96. Zhang, X.-W., et al., *Chinese herbal medicine for advanced non-small-cell lung cancer: a systematic review and meta-analysis.* The American journal of Chinese medicine, 2018. **46**(05): p. 923-952.
97. Zhang, Y., et al., *Acupuncture for breast cancer: A systematic review and meta-analysis of patient-reported outcomes.* Frontiers in Oncology, 2021. **11**: p. 646315.
98. Zhang, Y., et al., *Effects of acupuncture on cancer-related fatigue: a meta-analysis.* Supportive Care in Cancer, 2018. **26**: p. 415-425.
99. Yueyang, Z., et al., *Effectiveness and safety of traditional Chinese medical therapy for cancer-related fatigue: A systematic review and Meta-analysis of randomized controlled trials.* Journal of Traditional Chinese Medicine, 2020. **40**(5): p. 738.
100. Zhu, X.-Y., et al., *Physical therapies for psychosomatic symptoms and quality of life induced by aromatase inhibitors in breast cancer patients: a systematic review and meta-analysis.* Frontiers in Oncology, 2021: p. 4703.
