# Supplementary material for: The Effects of Complementary Therapies on Patient-Reported Outcomes: An Overview of Recent Systematic Reviews in Oncology
Source: Cancers (Basel). 2023 Sep 11;15(18):4513. doi: 10.3390/cancers15184513 (PMC10526744; doi:10.3390/cancers15184513)
Supplement: Supplementary file 1 [file cancers-15-04513-s001.zip › Table S2 - AMSTAR table.pdf]

Supplemental material S3 – Quality appraisal

The seven critical domains of the AMSTAR 2 [1]	Key	
**Item 2**—Did the report of the review contain an explicit statement that the review methods were established prior to the conduct of the review and did the report justify any significant deviations from the protocol?<br>**Item 4**—Did the review authors use a comprehensive literature search strategy?<br>**Item 7**—Did the review authors provide a list of excluded studies and justify the exclusions?<br>**Item 9**—Did the review authors use a satisfactory technique for assessing the risk of bias (RoB) in individual studies that were included in the review?<br>**Item 11**—If meta-analysis was performed, did the review authors use appropriate methods for statistical combination of results?<br>**Item 13**—Did the review authors account for RoB in primary studies when interpreting/discussing the results of the review?<br>**Item 15**—If they performed quantitative synthesis did the review authors carry out an adequate investigation of publication bias (small study bias) and discuss its likely impact on the results of the review?	● (red)	Yes
	● (yellow)	Partial yes
	● (green)	No

Table S2.
AMSTAR 2 scoring for each included systematic review

First author, year, ref	Item 2	Item 4	Item 7	Item 9	Item 11	Item 13	Item 15
Armer 2020 [2]	Yes	Yes	Yes	No	No	Yes	Yes
Bai 2022 [3]	No	Partial yes	Yes	No	No	Yes	No
Bro 2018 [4]	No	Partial yes	Yes	No	Yes	Yes	Yes
Cai 2022 [5]	No	Partial yes	Yes	No	No	No	No
Chan 2021 [6]	No	Partial yes	Yes	No	No	No	No
Chang 2021 [7]	Yes	Partial yes	Yes	No	No	Yes	Yes
Chen 2018 [8]	Yes	Yes	Yes	No	Yes	Yes	Yes
Chen 2019 [9]	Yes	Partial yes	Yes	No	No	Yes	Yes
Chen 2020 [10]	No	Yes	Yes	No	Yes	Yes	Yes
Chen 2021 [11]	Yes	Partial yes	Yes	No	No	Yes	Yes
Chen 2021 [12]	No	Partial yes	Yes	No	No	Yes	No
Chien 2019 [13]	Yes	Partial yes	Yes	No	Yes	Yes	Yes
Chien 2020 [14]	Yes	Partial yes	Yes	No	Yes	Yes	Yes
Coutiño-Escamilla 2019 [15]	Yes	Partial yes	Yes	Yes	No	No	No
Dai 2021 [16]	No	Partial yes	Yes	No	No	Yes	No
Danon 2022 [17]	No	Yes	Yes	No	Yes	Yes	No
Deng 2018 [18]	No	Partial yes	Yes	No	No	No	No
Dong 2019 [19]	Yes	Partial yes	Yes	No	Yes	Yes	Yes
Dong 2021 [20]	Yes	Partial yes	Yes	No	No	No	Yes
El-Hashimi 2019 [21]	Yes	Partial yes	Yes	Yes	No	Yes	Yes

First author, year, ref	Item 2	Item 4	Item 7	Item 9	Item 11	Item 13	Item 15
Gao 2021 [22]	🟢	🟡	🔴	🟢	🔴	🔴	🔴
Gonzalez 2021 [23]	🟢	🟡	🔴	🟢	🟢	🔴	🟢
Haussmann 2022 [24]	🟢	🔴	🔴	🟢	🔴	🔴	🔴
Jin 2021 [25]	🔴	🟡	🔴	🟢	🟢	🔴	🟢
He 2020 [26]	🟢	🟡	🔴	🟢	🟢	🔴	🔴
He 2020 [27]	🔴	🟡	🔴	🟢	🔴	🔴	🔴
Hou 2020 [28]	🟢	🟡	🔴	🟢	🟢	🔴	🟢
Hsieh 2021 [29]	🟢	🟡	🔴	🟢	🔴	🟢	🟢
Hsueh 2021 [30]	🟢	🟢	🔴	🟢	🟢	🔴	🔴
Hu 2022 [31]	🟢	🟡	🔴	🟢	🟢	🔴	🔴
Huang 2020 [32]	🔴	🟡	🔴	🟢	🟢	🔴	🟢
Jang 2020 [33]	🟢	🟡	🔴	🟢	🔴	🔴	🔴
Jang 2020 [34]	🟢	🟡	🔴	🟢	🔴	🔴	🔴
Jihong 2021 [35]	🟢	🟡	🔴	🟢	🟢	🔴	🔴
Jin 2020 [36]	🟢	🟡	🔴	🟢	🟢	🔴	🔴
Jing 2018 [37]	🟢	🟡	🔴	🟢	🟢	🔴	🔴
Kannan 2022 [38]	🟢	🟡	🟢	🟡	🔴	🔴	🔴
Kuo 2021 [39]	🔴	🟡	🔴	🟢	🔴	🔴	🔴
Kwon 2021 [40]	🟢	🟡	🟢	🟢	🟢	🔴	🔴
Li 2019 [41]	🟢	🟡	🔴	🟢	🟢	🔴	🔴
Li 2019 [42]	🟢	🟡	🔴	🟢	🟢	🔴	🟢
Li 2020 [43]	🟢	🟡	🔴	🟢	🟢	🔴	🔴
Li 2020 [44]	🔴	🟡	🔴	🟢	🟢	🔴	🔴
Li 2020 [45]	🔴	🟡	🔴	🟢	🟢	🔴	🔴
Li 2021 [46]	🔴	🟡	🔴	🟢	🔴	🔴	🔴
Li 2021 [47]	🔴	🟡	🔴	🟢	🟢	🔴	🔴
Li 2021 [48]	🔴	🟡	🔴	🟢	🔴	🔴	🔴
Lin 2019 [49]	🟢	🟡	🔴	🟢	🟢	🔴	🟢
Lin 2022 [50]	🟢	🟡	🔴	🟢	🟢	🔴	🔴

First author, year, ref	Item 2	Item 4	Item 7	Item 9	Item 11	Item 13	Item 15
Lin 2022 [51]	🔴	🟡	🔴	🟢	🟢	🔴	🔴
Liu 2019 [52]	🔴	🔴	🔴	🟢	🟢	🔴	🟢
Liu 2020 [53]	🔴	🟢	🔴	🟡	🟢	🔴	🔴
Liu 2020 [54]	🟢	🟡	🔴	🟢	🔴	🔴	🔴
Liu 2021 [55]	🟢	🟡	🔴	🟢	🟢	🔴	🔴
Liu 2021 [56]	🔴	🟡	🔴	🟢	🟢	🔴	🔴
Lu 2021 [57]	🟢	🟡	🔴	🟢	🟢	🔴	🔴
Luo 2020 [58]	🟢	🟡	🔴	🟢	🟢	🔴	🔴
Ma 2019 [59]	🔴	🟡	🔴	🟢	🟢	🔴	🔴
Mai 2022 [60]	🔴	🟡	🔴	🟢	🟢	🔴	🟢
Ni 2019 [61]	🔴	🟡	🔴	🟢	🔴	🔴	🔴
Ni 2020 [62]	🟢	🟡	🔴	🟢	🟢	🔴	🔴
O'Neill 2020 [63]	🔴	🟡	🔴	🟢	🟢	🔴	🔴
PinheirodaSilva 2019 [64]	🟢	🟡	🔴	🟡	🟢	🟢	🔴
Qi 2021 [65]	🔴	🟡	🔴	🟢	🟢	🔴	🟢
Qiao 2022 [66]	🔴	🟡	🔴	🟢	🟢	🔴	🟢
Schell 2019 [67]	🟢	🟡	🟢	🟢	🔴	🟢	🔴
She 2021 [68]	🔴	🟢	🔴	🟢	🟢	🔴	🟢
Shi 2021 [69]	🔴	🟡	🔴	🟢	🔴	🔴	🔴
Sinha 2021 [70]	🟢	🟡	🔴	🟢	🔴	🔴	🔴
Song 2018 [71]	🟢	🟡	🔴	🟢	🟢	🔴	🟢
Suh 2021 [72]	🟢	🟡	🔴	🟢	🔴	🔴	🔴
Tan 2021 [73]	🟢	🟡	🔴	🟢	🟢	🔴	🔴
Tang 2019 [74]	🔴	🟡	🔴	🟢	🟢	🔴	🟢
Wang 2018 [75]	🔴	🟡	🔴	🟢	🔴	🔴	🔴
Wang 2019 [76]	🔴	🟡	🔴	🟢	🔴	🔴	🔴
Wang 2020 [77]	🔴	🟡	🔴	🟢	🔴	🔴	🔴
Wang 2020 [78]	🔴	🟡	🔴	🟢	🟢	🟢	🟢

First author, year, ref	Item 2	Item 4	Item 7	Item 9	Item 11	Item 13	Item 15
Wang 2021 [79]	🟢	🟡	🔴	🟢	🟢	🔴	🟢
Wang 2021 [80]	🟢	🟡	🔴	🟢	🟢	🔴	🔴
Wang 2021 [81]	🔴	🟡	🔴	🟢	🟢	🔴	🟢
Wu 2019 [82]	🔴	🟡	🔴	🟢	🟢	🔴	🔴
Xie 2020 [83]	🔴	🟡	🔴	🟢	🟢	🔴	🔴
Xu 2021 [84]	🟢	🟡	🔴	🟢	🔴	🔴	🔴
Xunlin 2020 [85]	🟢	🔴	🔴	🟢	🔴	🔴	🔴
Yang 2020 [86]	🔴	🔴	🔴	🟢	🔴	🔴	🔴
Yang 2021 [87]	🔴	🟡	🔴	🟢	🔴	🔴	🔴
YangözŞ 2019 [88]	🔴	🟡	🔴	🟢	🔴	🔴	🟢
Yao 2022 [89]	🟢	🟡	🔴	🟢	🟢	🟢	🟢
Yi 2021 [90]	🔴	🟡	🔴	🟢	🟢	🔴	🔴
Yin 2020 [91]	🔴	🟡	🔴	🟢	🟢	🔴	🔴
Yoon 2021 [92]	🟢	🟡	🔴	🟢	🟢	🔴	🟢
Yuanqing 2020 [93]	🔴	🟡	🔴	🟢	🟢	🔴	🔴
Zeng 2020 [94]	🔴	🟡	🔴	🟢	🔴	🔴	🟢
Zeng 2022 [95]	🔴	🟡	🔴	🟢	🟢	🔴	🟢
Zhang 2018 [96]	🔴	🟡	🔴	🟢	🟢	🔴	🟢
Zhang 2018 [97]	🟢	🟡	🟢	🟢	🟢	🟢	🔴
Zhang 2018 [98]	🔴	🟡	🔴	🟢	🟢	🔴	🟢
Zhang 2021 [99]	🟢	🟡	🔴	🟢	🔴	🔴	🔴
Zhao 2020 [100]	🟢	🟡	🔴	🟢	🔴	🔴	🔴
Zhu 2021 [101]	🔴	🟡	🔴	🟢	🟢	🔴	🔴

References

1. Shea, B.J., et al., *AMSTAR 2: a critical appraisal tool for systematic reviews that include randomised or non-randomised studies of healthcare interventions, or both.* bmj, 2017. **358**.
2. Armer, J.S. and S.K. Lutgendorf, *The impact of yoga on fatigue in cancer survivorship: a meta-analysis.* JNCI cancer spectrum, 2020. **4**(2): p. pkz098.

3. Bai, X., et al., *Effects of integrated Chinese traditional medicine and conventional western medicine on the quality of life of breast cancer patients: a systematic review and meta-analysis.* Evidence-Based Complementary and Alternative Medicine, 2022. **2022**.
4. Bro, M.L., et al., *Kind of blue: A systematic review and meta-analysis of music interventions in cancer treatment.* Psycho-oncology, 2018. **27**(2): p. 386-400.
5. Cai, Q., et al., *Tai Chi for anxiety and depression symptoms in cancer, stroke, heart failure, and chronic obstructive pulmonary disease: A systematic review and meta-analysis.* Complementary Therapies in Clinical Practice, 2022. **46**: p. 101510.
6. Chan, Y.-T., et al., *Systematic Review with Meta-Analysis: Effectiveness and Safety of Acupuncture as Adjuvant Therapy for Side Effects Management in Drug Therapy-Receiving Breast Cancer Patients.* Evidence-Based Complementary and Alternative Medicine, 2021. **2021**.
7. Chang, Y.-C., et al., *Short-term effects of randomized mindfulness-based intervention in female breast cancer survivors: a systematic review and meta-analysis.* Cancer Nursing, 2021. **44**(6): p. E703-E714.
8. Chen, C.-Y., X.-X. Lin, and X. Wang, *Efficacy of non-invasive auricular acupressure for treating constipation in leukemia patients undergoing chemotherapy: a systematic review.* Complementary Medicine Research, 2018. **25**(6): p. 406-412.
9. Chen, Z.-Q., et al., *Chinese herbal medicine for epidermal growth factor receptor inhibitor-induced skin rash in patients with malignancy: An updated meta-analysis of 23 randomized controlled trials.* Complementary Therapies in Medicine, 2019. **47**: p. 102167.
10. Chen, S., et al., *Efficacy and safety of TCM combined with chemotherapy for SCLC: a systematic review and meta-analysis.* Journal of Cancer Research and Clinical Oncology, 2020. **146**: p. 2913-2935.
11. Chen, L., et al., *Efficacy of auricular acupressure in prevention and treatment of chemotherapy-induced nausea and vomiting in patients with cancer: a systematic review and meta-analysis.* Evidence-Based Complementary and Alternative Medicine, 2021. **2021**: p. 1-11.
12. Chen, Y., et al., *Efficacy of Herbal Medicines Intervention for Colorectal Cancer Patients With Chemotherapy-Induced Gastrointestinal Toxicity—a Systematic Review and Meta-Analysis.* Frontiers in Oncology, 2021. **11**: p. 629132.
13. Chien, T.-J., et al., *The efficacy of acupuncture in chemotherapy-induced peripheral neuropathy: systematic review and meta-analysis.* Integrative cancer therapies, 2019. **18**: p. 1534735419886662.
14. Chien, T.-J., et al., *The maintenance effect of acupuncture on breast cancer-related menopause symptoms: a systematic review.* Climacteric, 2020. **23**(2): p. 130-139.
15. Coutiño-Escamilla, L., et al., *Non-pharmacological therapies for depressive symptoms in breast cancer patients: Systematic review and meta-analysis of randomized clinical trials.* The Breast, 2019. **44**: p. 135-143.
16. Dai, L., et al., *Acupuncture and derived therapies for pain in palliative cancer management: systematic review and meta-analysis based on single-arm and controlled trials.* Journal of Palliative Medicine, 2021. **24**(7): p. 1078-1099.
17. Danon, N., et al., *Are mind–body therapies effective for relieving cancer-related pain in adults? A systematic review and meta-analysis.* Psycho-Oncology, 2022. **31**(3): p. 345-371.
18. Deng, B. and W. Sun, *Herbal medicine for hand–foot syndrome induced by fluoropyrimidines: A systematic review and meta-analysis.* Phytotherapy Research, 2018. **32**(7): p. 1211-1228.
19. Dong, B., et al., *Yoga has a solid effect on cancer-related fatigue in patients with breast cancer: a meta-analysis.* Breast cancer research and treatment, 2019. **177**: p. 5-16.
20. Dong, B., et al., *Wrist-ankle acupuncture has a positive effect on cancer pain: a meta-analysis.* BMC complementary medicine and therapies, 2021. **21**(1): p. 1-10.
21. El-Hashimi, D. and K.M. Gorey, *Yoga-specific enhancement of quality of life among women with breast cancer: systematic review and exploratory meta-analysis of randomized*

controlled trials. Journal of evidence-based integrative medicine, 2019. **24**: p. 2515690X19828325.
22. Gao, Y., et al., *Effects of Acupuncture and Moxibustion on Breast Cancer-Related Lymphedema: A Systematic Review and Meta-Analysis of Randomized Controlled Trials.* Integrative Cancer Therapies, 2021. **20**: p. 15347354211044107.
23. Gonzalez, M., et al., *Yoga for depression and anxiety symptoms in people with cancer: a systematic review and meta-analysis.* Psycho-Oncology, 2021. **30**(8): p. 1196-1208.
24. Haussmann, A., et al., *Meta-analysis of randomized controlled trials on yoga, psychosocial, and mindfulness-based interventions for cancer-related fatigue: what intervention characteristics are related to higher efficacy?* Cancers, 2022. **14**(8): p. 2016.
25. Jin, H., et al., *Traditional herbal medicine combined with first-line platinum-based chemotherapy for advanced non-small-cell lung cancer: A PRISMA-compliant systematic review and meta-analysis.* Medicine, 2021. **100**(37).
26. He, Y., et al., *Clinical evidence for association of acupuncture and acupressure with improved cancer pain: a systematic review and meta-analysis.* JAMA oncology, 2020. **6**(2): p. 271-278.
27. He, J., et al., *Mindfulness ased stress reduction interventions for cancer related fatigue: a meta-analysis and systematic review.* Journal of the National Medical Association, 2020. **112**(4): p. 387-394.
28. Hou, X.-B. and D.-D. Chen, *Effectiveness and safety of acupuncture on cancer pain: a meta-analysis.* TMR Integrative Medicine, 2020. **4**: p. e20018.
29. Hsieh, S.-H., et al., *The effect of acupressure on relieving cancer-related fatigue: a systematic review and meta-analysis of randomized controlled trials.* Cancer Nursing, 2021. **44**(6): p. E578-E588.
30. Hsueh, E.-J., et al., *Effects of yoga on improving quality of life in patients with breast cancer: a meta-analysis of randomized controlled trials.* Breast Cancer, 2021. **28**: p. 264-276.
31. Hu, J., et al., *Clinical efficacy and safety of traditional medicine preparations combined with chemotherapy for advanced pancreatic cancer: A systematic review and meta-analysis.* Frontiers in Oncology, 2022. **12**.
32. Huang, Z., et al., *Effect of traditional Chinese medicine injection on cancer-related fatigue: A meta-analysis based on existing evidence.* Evidence-Based Complementary and Alternative Medicine, 2020. **2020**.
33. Jang, S., et al., *Acupuncture as an adjuvant therapy for management of treatment-related symptoms in breast cancer patients: systematic review and meta-analysis (PRISMA-compliant).* Medicine, 2020. **99**(50).
34. Jang, A., et al., *The effects of acupuncture on cancer-related fatigue: updated systematic review and meta-analysis.* Integrative cancer therapies, 2020. **19**: p. 1534735420949679.
35. Song, J., *The effectiveness of yoga on cancer-related fatigue: a systematic review and meta-analysis.* Number 2/March 2021, 2021. **48**(2): p. 207-228.
36. Jin, Y., et al., *Efficacy and safety of acupuncture against chemotherapy-induced peripheral neuropathy: a systematic review and meta-analysis.* Evidence-Based Complementary and Alternative Medicine, 2020. **2020**.
37. Jing, X., et al., *Auricular acupressure is an alternative in treating constipation in leukemia patients undergoing chemotherapy: A systematic review and meta-analysis.* Complementary Therapies in Clinical Practice, 2018. **31**: p. 282-289.
38. Kannan, P., et al., *Efficacy of physical therapy interventions on quality of life and upper quadrant pain severity in women with post-mastectomy pain syndrome: a systematic review and meta-analysis.* Quality of Life Research, 2022. **31**(4): p. 951-973.
39. Kuo, C.-C., et al., *Clinical effects of Baduanjin Qigong exercise on cancer patients: a systematic review and meta-analysis on randomized controlled trials.* Evidence-Based Complementary and Alternative Medicine, 2021. **2021**: p. 1-10.

40. Kwon, C.Y., et al., *Effectiveness and safety of herbal medicine for cancer-related fatigue in lung cancer survivors: A systematic review and meta-analysis.* Phytotherapy Research, 2021. **35**(2): p. 751-770.
41. Li, M., et al., *Twelve Chinese herbal preparations for the treatment of depression or depressive symptoms in cancer patients: a systematic review and meta-analysis of randomized controlled trials.* BMC complementary and alternative medicine, 2019. **19**(1): p. 1-16.
42. Li, Z., et al., *The method of activating blood and dredging collaterals for reducing chemotherapy-induced peripheral neuropathy: a systematic review and meta-analysis.* Evidence-Based Complementary and Alternative Medicine, 2019. **2019**.
43. Li, S., et al., *Chinese herbal medicine for reducing chemotherapy-associated side-effects in breast cancer patients: a systematic review and meta-analysis.* Frontiers in Oncology, 2020. **10**: p. 599073.
44. Li, Y., et al., *The effectiveness of music therapy for patients with cancer: A systematic review and meta-analysis.* Journal of Advanced Nursing, 2020. **76**(5): p. 1111-1123.
45. Li, Y., et al., *Meta-analysis of paclitaxel-based chemotherapy combined with traditional Chinese medicines for gastric cancer treatment.* Frontiers in Pharmacology, 2020. **11**: p. 132.
46. Li, D.-h., et al., *Acupuncture combined with three-step analgesic drug therapy for treatment of cancer pain: a systematic review and meta-analysis of randomised clinical trials.* Evidence-based Complementary and Alternative Medicine, 2021. **2021**: p. 1-12.
47. Li, L., et al., *Evidence on efficacy and safety of Chinese medicines combined western medicines treatment for breast cancer with endocrine therapy.* Frontiers in Oncology, 2021. **11**: p. 661925.
48. Li, H., et al., *Acupuncture improves multiple treatment-related symptoms in breast cancer survivors: A systematic review and meta-analysis.* The Journal of Alternative and Complementary Medicine, 2021. **27**(12): p. 1084-1097.
49. Lin, W.-F., et al., *Efficacy of complementary and integrative medicine on health-related quality of life in cancer patients: a systematic review and meta-analysis.* Cancer management and research, 2019. **11**: p. 6663.
50. Lin, Y., et al., *Manual lymphatic drainage for breast cancer-related lymphedema: a systematic review and meta-analysis of randomized controlled trials.* Clinical Breast Cancer, 2022.
51. Lin, L.-Y., et al., *Effects of mindfulness-based therapy for cancer patients: A systematic review and meta-analysis.* Journal of Clinical Psychology in Medical Settings, 2022. **29**(2): p. 432-445.
52. Liu, Y., et al., *Integrative herbal medicine for chemotherapy-induced peripheral neuropathy and hand-foot syndrome in colorectal cancer: a systematic review and meta-analysis.* Integrative cancer therapies, 2019. **18**: p. 1534735418817833.
53. Liu, L., et al., *The effectiveness of tai chi in breast cancer patients: A systematic review and meta-analysis.* Complementary Therapies in Clinical Practice, 2020. **38**: p. 101078.
54. Liu, J., et al., *Nonhormonal hot flash management for breast cancer survivors: a systematic review and network meta-analysis.* Evidence-Based Complementary and Alternative Medicine, 2020. **2020**.
55. Liu, C., et al., *A meta-analysis: intervention effect of mind-body exercise on relieving cancer-related fatigue in breast cancer patients.* Evidence-Based Complementary and Alternative Medicine, 2021. **2021**.
56. Liu, X., et al., *Acupuncture for arthralgia induced by aromatase inhibitors in patients with breast cancer: a systematic review and meta-analysis.* Integrative Cancer Therapies, 2021. **20**: p. 1534735420980811.
57. Lu, Y., et al., *Chinese herbal medicine combined with first-generation EGFR-TKIs in treatment of advanced non-small cell lung cancer with EGFR sensitizing mutation: a systematic review and meta-analysis.* Frontiers in Pharmacology, 2021. **12**: p. 698371.
58. Luo, X.-C., et al., *Effect of Tai Chi Chuan in breast cancer patients: a systematic review and meta-analysis.* Frontiers in Oncology, 2020. **10**: p. 607.

59. Ma, H.-L., et al., *The effectiveness of moxibustion for cancer-related fatigue: An updated systematic review and meta-analysis.* European Journal of Integrative Medicine, 2019. **30**: p. 100960.
60. Mai, Q., et al., *Effects of acupressure on cancer-related pain management: A systematic review and meta-analysis of randomized controlled trials.* European Journal of Integrative Medicine, 2022: p. 102120.
61. Ni, X., et al., *The effects of Tai Chi on quality of life of cancer survivors: a systematic review and meta-analysis.* Supportive Care in Cancer, 2019. **27**: p. 3701-3716.
62. Ni, X., et al., *Acupuncture for radiation-induced xerostomia in cancer patients: a systematic review and meta-analysis.* Integrative Cancer Therapies, 2020. **19**: p. 1534735420980825.
63. O'Neill, M., et al., *The effect of yoga interventions on cancer-related fatigue and quality of life for women with breast cancer: a systematic review and meta-analysis of randomized controlled trials.* Integrative cancer therapies, 2020. **19**: p. 1534735420959882.
64. da Silva, F.P., et al., *Manual therapy as treatment for chronic musculoskeletal pain in female breast cancer survivors: a systematic review and meta-analysis.* Journal of Manipulative and Physiological Therapeutics, 2019. **42**(7): p. 503-513.
65. Qi, Y., et al., *Music interventions can alleviate cancer-related fatigue: a metaanalysis.* Supportive Care in Cancer, 2021. **29**: p. 3461-3470.
66. Qiao, J., et al., *Effect of Manual Lymphatic Drainage on Breast Cancer–Related Postmastectomy Lymphedema: A Meta-analysis of Randomized Controlled Trials.* Cancer Nursing, 2023. **46**(2): p. 159-166.
67. Schell, L.K., et al., *Mindfulness-based stress reduction for women diagnosed with breast cancer.* Cochrane Database of Systematic Reviews, 2019(3).
68. She, Y., et al., *the therapeutic principle of combined strengthening Qi and eliminating pathogens in treating middle-advanced primary liver cancer: a systematic review and meta-analysis.* Frontiers in Pharmacology, 2021: p. 2783.
69. Shi, G., et al., *A systematic review and meta-analysis of traditional Chinese medicine with chemotherapy in breast cancer.* Gland Surgery, 2021. **10**(5): p. 1744.
70. Sinha, M.K., et al., *Progressive muscle relaxation and guided imagery in breast cancer: a systematic review and meta-analysis of randomised controlled trials.* Indian Journal of Palliative Care, 2021. **27**(2): p. 336.
71. Song, S., et al., *Ameliorative effects of Tai Chi on cancer-related fatigue: a meta-analysis of randomized controlled trials.* Supportive Care in Cancer, 2018. **26**: p. 2091-2102.
72. Suh, H.-W., et al., *The mindfulness-based stress reduction program for improving sleep quality in cancer survivors: a systematic review and meta-analysis.* Complementary Therapies in Medicine, 2021. **57**: p. 102667.
73. Tan, J.-Y.B., et al., *Acupoint stimulation for cancer-related fatigue: A quantitative synthesis of randomised controlled trials.* Complementary Therapies in Clinical Practice, 2021. **45**: p. 101490.
74. Tang, M.-F., et al., *Walking is more effective than yoga at reducing sleep disturbance in cancer patients: A systematic review and meta-analysis of randomized controlled trials.* Sleep medicine reviews, 2019. **47**: p. 1-8.
75. Wang, X.-P., et al., *Acupuncture for the relief of hot flashes in breast cancer patients: A systematic review and meta-analysis of randomized controlled trials and observational studies.* Journal of cancer research and therapeutics, 2018. **14**(Suppl 3): p. S600-S608.
76. Wang, R., et al., *Efficacy and safety of Chinese herbal medicine on ovarian cancer after reduction surgery and adjuvant chemotherapy: a systematic review and meta-analysis.* Frontiers in Oncology, 2019. **9**: p. 730.
77. Wang, L.-C., et al., *Systematic review and meta-analysis of Chinese herbal medicine as adjuvant treatment in advanced non-small cell lung cancer patients.* Complementary Therapies in Medicine, 2020. **52**: p. 102472.

78. Wang, W.-L., et al., *The effect of yoga on sleep quality and insomnia in women with sleep problems: a systematic review and meta-analysis.* BMC psychiatry, 2020. **20**: p. 1-19.
79. Wang, R., et al., *Efficacy of qigong exercise for treatment of fatigue: a systematic review and meta-analysis.* Frontiers in medicine, 2021. **8**: p. 684058.
80. Wang, Y., et al., *Auricular acupressure therapy for patients with cancer with sleep disturbance: a systematic review and meta-analysis.* Evidence-Based Complementary and Alternative Medicine, 2021. **2021**.
81. Wang, Y.-h., J.-y. Chang, and L. Feng, *Effect of oral Chinese medicine combined with Western medicine on cancer pain: a meta-analysis.* Chinese journal of integrative medicine, 2021. **27**: p. 713-720.
82. Wu, J., et al., *Traditional Chinese medicine preparation combined therapy may improve chemotherapy efficacy: a systematic review and meta-analysis.* Evidence-Based Complementary and Alternative Medicine, 2019. **2019**.
83. Xie, C., et al., *Mindfulness-based stress reduction can alleviate cancer-related fatigue: a meta-analysis.* Journal of psychosomatic research, 2020. **130**: p. 109916.
84. Xu, G., et al., *Acupuncture for Quality of Life of Patients with Defecation Dysfunction after Sphincter Preserving Surgery for Rectal Cancer: A Systematic Review.* Evidence-Based Complementary and Alternative Medicine, 2021. **2021**.
85. Xunlin, N., Y. Lau, and P. Klainin-Yobas, *The effectiveness of mindfulness-based interventions among cancer patients and survivors: a systematic review and meta-analysis.* Supportive Care in Cancer, 2020. **28**: p. 1563-1578.
86. Yang, J., et al., *Efficacy of traditional Chinese Medicine combined with chemotherapy in patients with non-small cell lung cancer (NSCLC): a meta-analysis of randomized clinical trials.* Supportive Care in Cancer, 2020. **28**: p. 3571-3579.
87. Yang, T., et al., *Effectiveness of five-element music therapy in cancer patients: A systematic review and meta-analysis.* Complementary Therapies in Clinical Practice, 2021. **44**: p. 101416.
88. Yangöz, Ş.T. and Z. Özer, *The effect of music intervention on patients with cancer-related pain: A systematic review and meta-analysis of randomized controlled trials.* Journal of Advanced Nursing, 2019. **75**(12): p. 3362-3373.
89. Yao, Z., et al., *Moxibustion for alleviating chemotherapy-induced gastrointestinal adverse effects: a systematic review of randomized controlled trials.* Complementary Therapies in Clinical Practice, 2022: p. 101527.
90. Yi, L.-J., et al., *Effects of yoga on health-related quality, physical health and psychological health in women with breast cancer receiving chemotherapy: a systematic review and meta-analysis.* Ann Palliat Med, 2021. **10**(2): p. 1961-1975.
91. Yin, J., L. Tang, and R.K. Dishman, *The efficacy of Qigong practice for cancer-related fatigue: A systematic review and meta-analysis of randomized controlled trials.* Mental Health and Physical Activity, 2020. **19**: p. 100347.
92. Yoon, J.-H., et al., *Traditional herbal medicine for insomnia in patients with cancer: a systematic review and meta-analysis.* Frontiers in Pharmacology, 2021. **12**: p. 753140.
93. Yuanqing, P., et al., *Acupuncture for Hormone Therapy–Related Side Effects in Breast Cancer Patients: A GRADE-Assessed Systematic Review and Updated Meta-Analysis.* Integrative cancer therapies, 2020. **19**: p. 1534735420940394.
94. Zeng, Y., et al., *Nonpharmacological interventions for cancer-related cognitive impairment in adult cancer patients: a network meta-analysis.* International journal of nursing studies, 2020. **104**: p. 103514.
95. Zeng, J., et al., *Effect of hypnosis before general anesthesia on postoperative outcomes in patients undergoing minor surgery for breast cancer: a systematic review and meta-analysis.* Gland Surgery, 2022. **11**(3): p. 588.
96. Zhang, Y., et al., *Effects of acupuncture on cancer-related fatigue: a meta-analysis.* Supportive Care in Cancer, 2018. **26**: p. 415-425.

97. Zhang, H.W., et al., *Moxibustion for alleviating side effects of chemotherapy or radiotherapy in people with cancer.* Cochrane Database of Systematic Reviews, 2018(11).
98. Zhang, X.-W., et al., *Chinese herbal medicine for advanced non-small-cell lung cancer: a systematic review and meta-analysis.* The American journal of Chinese medicine, 2018. **46**(05): p. 923-952.
99. Zhang, Y., et al., *Acupuncture for breast cancer: A systematic review and meta-analysis of patient-reported outcomes.* Frontiers in Oncology, 2021. **11**: p. 646315.
100. Yueyang, Z., et al., *Effectiveness and safety of traditional Chinese medical therapy for cancer-related fatigue: A systematic review and Meta-analysis of randomized controlled trials.* Journal of Traditional Chinese Medicine, 2020. **40**(5): p. 738.
101. Zhu, X.-Y., et al., *Physical therapies for psychosomatic symptoms and quality of life induced by aromatase inhibitors in breast cancer patients: a systematic review and meta-analysis.* Frontiers in Oncology, 2021: p. 4703.
